# Neural mechanisms of awareness of action

**DOI:** 10.1093/pnasnexus/pgag220

**Published:** 2026-07-02

**Authors:** David S Jin, Oumayma Agdali, Taruna Yadav, Sharif I Kronemer, Sydney Kunkler, Shweta Majumder, Maya Khurana, Marie McCusker, Ivory Fu, Emily J Siff, Aya Khalaf, Kate L Christison-Lagay, Shanae L Aerts, Qilong Xin, Jing-Jing Li, Sarah H McGill, Michael J Crowley, Hal Blumenfeld

**Affiliations:** Department of Neurology, Yale University School of Medicine, New Haven, CT 06520, USA; Interdepartmental Neuroscience Program, Yale University, New Haven, CT 06520, USA; Department of Neurology, Yale University School of Medicine, New Haven, CT 06520, USA; Interdepartmental Neuroscience Program, Yale University, New Haven, CT 06520, USA; Department of Neurology, Yale University School of Medicine, New Haven, CT 06520, USA; Department of Neurology, Yale University School of Medicine, New Haven, CT 06520, USA; Interdepartmental Neuroscience Program, Yale University, New Haven, CT 06520, USA; Department of Neurology, Yale University School of Medicine, New Haven, CT 06520, USA; Department of Neurology, Yale University School of Medicine, New Haven, CT 06520, USA; Department of Neurology, Yale University School of Medicine, New Haven, CT 06520, USA; Department of Neurology, Yale University School of Medicine, New Haven, CT 06520, USA; Interdepartmental Neuroscience Program, Yale University, New Haven, CT 06520, USA; Department of Neurology, Yale University School of Medicine, New Haven, CT 06520, USA; Department of Neurology, Yale University School of Medicine, New Haven, CT 06520, USA; Interdepartmental Neuroscience Program, Yale University, New Haven, CT 06520, USA; Department of Neurology, Yale University School of Medicine, New Haven, CT 06520, USA; Department of Neurology, Yale University School of Medicine, New Haven, CT 06520, USA; Department of Neurology, Yale University School of Medicine, New Haven, CT 06520, USA; Interdepartmental Neuroscience Program, Yale University, New Haven, CT 06520, USA; Department of Neurology, Yale University School of Medicine, New Haven, CT 06520, USA; Interdepartmental Neuroscience Program, Yale University, New Haven, CT 06520, USA; Department of Neurology, Yale University School of Medicine, New Haven, CT 06520, USA; Department of Neurology, Yale University School of Medicine, New Haven, CT 06520, USA; Interdepartmental Neuroscience Program, Yale University, New Haven, CT 06520, USA; Child Study Center, Yale University School of Medicine, New Haven, CT 06519, USA; Department of Neurology, Yale University School of Medicine, New Haven, CT 06520, USA; Interdepartmental Neuroscience Program, Yale University, New Haven, CT 06520, USA; Department of Neuroscience, Yale University School of Medicine, New Haven, CT 06520, USA; Department of Neurosurgery, Yale University School of Medicine, New Haven, CT 06520, USA

**Keywords:** volition, consciousness, attention, perception, event-related potentials

## Abstract

Awareness of action (AoA), or conscious awareness of an action just performed, is an important part of daily experience with major practical and ethical relevance, yet the neural mechanisms of AoA remain largely unknown. The main barrier to studying AoA is a lack of experimental paradigms to directly compare neural activity in aware versus unaware actions. Borrowing from the field of perceptual awareness, where exciting progress has been made by contrastive analysis of aware versus unaware stimuli, we developed a game where participants repeatedly perform nearly identical moves while engaged in a distractor task, and the participants then report awareness or unawareness of the moves they just performed. We found that on short timescales, aware actions had larger neurophysiological signals both preceding and following movement. The differences included both volitional and perceptual event-related potentials (premovement positivity, N140, and P300), as well as frontal midline theta, event-related alpha/beta desynchronization, and postmove blink rates. On longer time scales, we identified a novel positive event-related potential only preceding unaware moves and found behavioral and pupillometric evidence for decreased attention and arousal over minutes concurrent with AoA loss. Our findings reveal three neural mechanisms that may synergistically contribute to AoA: (i) long-term increases in arousal/attentional state at time of action; (ii) increased action-related motor volitional signals; and (iii) increased action-related sensory perceptual signals. Deeper understanding of AoA may ultimately elucidate the causes of variable AoA in daily life and lead to better treatments for impaired AoA in neuropsychiatric disorders.

Significance Statement Humans are often consciously aware of what we do in the world, but at times we are not. A common example is highway hypnosis, when immediately after driving a complex route we may not know the details, feeling as if on autopilot. This research study found that retrospective awareness of our actions is related to increased brain activity for arousal, motor performance, and sensory perception. Understanding how brain activity differs for aware versus unaware actions sheds light on a long-standing mystery of human behavior and may help treat brain disorders where awareness of action is lost.

## Introduction

Recent work has greatly advanced our understanding of the neural mechanisms of conscious awareness (1–5). However, the vast majority of this work has focused on sensory perceptual awareness rather than on awareness of motor actions. The neural origins of awareness of action (AoA), the ability to report an action just performed, have long been poorly understood, yet have been debated from the inception of psychological science. The loss of AoA is a part of everyday experience, seen in phenomena like “highway hypnosis” and “white line fever” where awareness of recent driving actions are lost, or being unable to know whether everyday tasks have been completed such as locking the door or turning off the gas (6, 7). Aberrant AoA is regularly seen in medical conditions such as Parkinson's disease, ataxia, and schizophrenia (8, 9). Despite the lack of understanding of neural mechanisms of AoA, definitions of AoA are also considered in legal judgments, with distinctions between murder versus manslaughter and voluntary versus involuntary crimes, depending on whether the offenses were committed with intention and awareness (10–12).

The early founders of modern psychological science fell into two primary camps on the origins of awareness of motor action, creating the “Two Williams Debate.” The first camp, led by Wilhelm Wundt, posited that the contents of action are represented a priori and that AoA is a mental, generative phenomenon independent of somatosensory feedback. The second camp, led by William James, suggested that the contents of action are represented a posteriori and that the somatosensory, afferent processes allow for AoA (13). We posit that neither perspective fully encapsulates the neural processes underlying AoA. Volitional as well as perceptual neural mechanisms both preaction and postaction may be needed to establish AoA on short-time scales, while additional state-related attention and arousal mechanisms could influence AoA over longer time periods.

To test whether AoA is an a priori or a posteriori process (Two Williams Debate) or whether other mechanisms may contribute, a carefully designed, behavioral paradigm to isolate instances of awareness or unawareness is needed. While previous studies of action have included varying motor components such as button presses when the participant feels the urge to move (e.g. the classic Libet paradigm), forced choices, and even bungee jumping, they do not query whether the participants are aware of the identity of the action or not (14–16). Therefore, they have not tested AoA itself, by looking for neural signals seen when AoA is present versus absent in a controlled setting. To address this, we developed a paradigm based upon a classic sliding block puzzle game, which participants complete at a self-set pace. Periodically, participants are asked questions about their just-completed move, to assess retrospective awareness of the previous action. This approach, referred to as contrastive analysis, where neural signals are compared between very similar events with versus without conscious awareness, has been applied widely to sensory paradigms but has not been used so far to study motor awareness (17–22). The paradigm we developed, involving repeated relatively similar clicks and keypresses, allows us to perform a contrastive method to AoA and to observe both preaction and postaction neural signals.

Should the neural mechanisms of AoA differ a priori, differences in neural activity are expected during the preparation and initiation of action. Previous studies of action, including the classic Libet paradigm, have established preaction differences in awareness of intent to act (23, 24). These studies have established the presence of the “bereitschaftspotential,” or readiness potential, a negativity in electroencephalogram (EEG) 1–2 s preceding the intent to act (15, 23–26). The readiness potential has been shown to be generated by activity in supplementary motor cortex and premotor cortex (27–30). Much closer to performance of the action (∼100 ms prior), a premovement positivity (PMP) has been observed as well, associated with the immediate initiation of the action as opposed to its planning (31–33). Additionally, preaction activation changes in prefrontal and parietal cortex have been shown to occur prior to intent to act, seen in both positron emission tomography and functional MRI (fMRI) (34–36). On the other hand, a posteriori differences in neural activity would manifest in perceptual signals, like those seen in the visual, auditory, and tactile domains. Known event-related potentials following perceptual stimuli include the face-specific N170, N100 (vision), auditory awareness negativity, and N140 (somatosensory awareness negativity) (17, 37, 38). In addition, signals which may support both a priori volitional and a posteriori perceptual hypotheses of AoA are known to exist. In the time-frequency domain, preaction beta frequency (12–30 Hz) suppression occurs prior to the initiation or imagination of a voluntary movement (39–41), and directives to attend to a visual stimulus are known to decrease the presence of beta activity (42). Lastly, suppression of alpha activity (8–12 Hz) has long been known to increase the probability of detecting a visual stimulus, and, like beta activity, is also quieted by the presentation of a visual stimulus (43–45) and is quieted prior to the execution of voluntary movement (46). Alpha suppression is also observed in auditory and tactile domains (47, 48), and alpha/beta event-related desynchronization more generally is viewed as signature of prominent behavioral or perceptual events and conscious awareness (49–51).

The view of action as purely a priori or a posteriori may also be incomplete. Actions in the real world are rarely performed as individual discretized motions, a drawback of many traditional forced choice and Libet-style studies. Instead, they are completed in sequence and under varying states of arousal. Studies of attentional states suggest that these flow states are bimodal in nature (i.e. “in the zone” or “out of the zone”), modulated by the default mode network, and can last on the order of seconds to minutes (52–54). We hypothesized that AoA may be lost more often during mind-wandering and daydreaming circumstances, generally states of lower-arousal or distraction from the task at hand. We encouraged these circumstances by having participants play the game while an engaging video appeared in the background, which they had to describe following each run. To measure the effects of fatigue and arousal-related factors on awareness over the course of the study, we used pupillometric measurements. Pupil diameter is a known to track with neural activity in the salience network and can provide rich, noninvasive information on arousal levels (55, 56). The success of multiple continuous performance task types can be predicted by pupil diameter (57). Decreased arousal in tandem with decreased AoA would provide concrete evidence for this anecdotal link.

We found clear differences in event-related potential, EEG time-frequency, and eye metric data both before and after action. We also found that arousal levels, measured by pupil diameter, decreased throughout the course of the experiment, in tandem with decreased levels of behavioral awareness and increased levels of unawareness. Thus, our findings support both hypotheses of the Two Williams Debate: that generative, volitional processes and afferent, perceptive processes allow for AoA. Our findings also highlight the role of longer-term attention and arousal states in the maintenance of AoA and that AoA must be considered in a broader context of sequences of actions.

## Results

### Behavioral results: task validation

**Figure 1:**
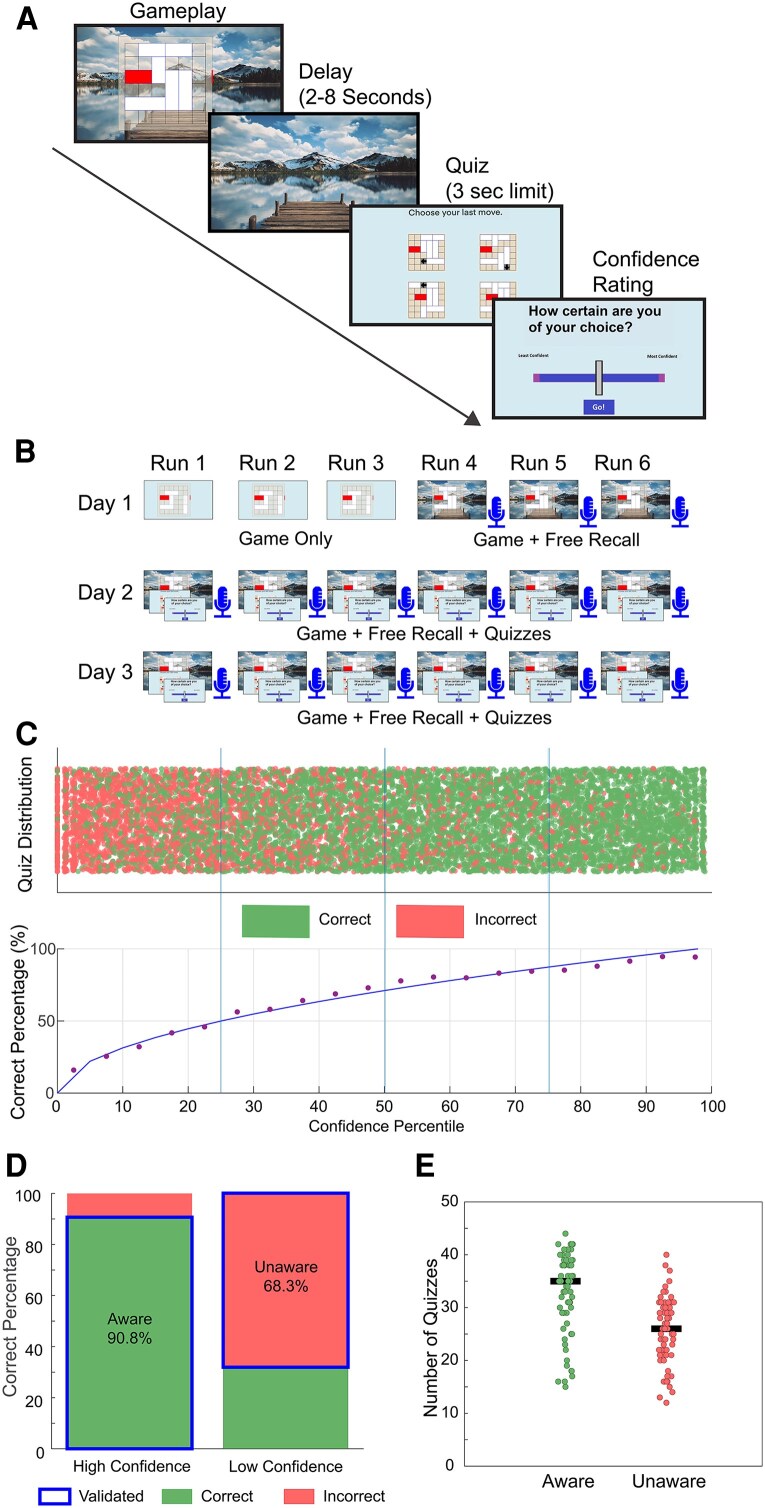
Experimental design and definition of aware and unaware trials. A) Experimental task. Participants played the task game by navigating a red block out of the bounds of the grid by moving obstructing blocks out of the way. Periodically, the game board would disappear for 2–8 s, and the following quiz could then be shown (after 50% of the board disappearances): (i) A multiple choice question in which participants selected the move just performed (direction and block shown by black arrows) and (ii) A sliding scale used to indicate their confidence in their choice. See also Supplementary Video S1 for example of game play. B) Experimental design schedule. The experiment consisted of one training day and two testing days. On day 1, participants first trained on a fixed set of puzzle configurations for three 10-min runs with the sliding block game only. Next, participants completed the sliding block puzzles while a background distractor video played for three runs. After each run, participants were asked to freely recall as many details as possible from the distractor video. The two testing days (days 2 and 3) were identical. Participants completed six runs while playing the slider game with the background distractor/recall task and with quizzes for sliding block game awareness administered periodically. C) All quiz results among all participants (*n* = 9,962 trials, in 67 participants). Confidence percentile is shown on the horizontal axis. Top panel shows all individual correct (green) and incorrect (red) trials on the multiple choice questions. Bottom panel shows percentage correct answers on the multiple choice questions for each 5% bin along the confidence percentile axis (same data as top panel). Confidence was divided into four quartiles. Trials with high confidence (>75th percentile), and validated by correct responses on the multiple choice quiz were defined as aware. Trials with low confidence (<25th percentile), validated by incorrect quiz responses were defined as unaware. D) Validation rates. Among high confidence (>75th percentile) responses, 90.8% were correct (expected: 100% correct), and among low confidence (<25th percentile) responses, 68.3% were incorrect (expected: 75% incorrect as the participant had a 1/4 chance of randomly guessing correctly). E) Participant aware and unaware trial totals. Participants (*n* = 67) attained a mean of 32.6 aware and 25.5 unaware trials (totals summed across both testing days). Participants who did not achieve at least 12 aware and at least 12 unaware trials were excluded. Horizontal black lines indicate mean values.

The goal of our behavioral task was to develop a paradigm where very similar aware and unaware actions occur repeatedly in a controlled setting. Such a paradigm, involving repeated relatively similar clicks and keypresses, would allow us to perform a contrastive method to AoA comparing neural signals for aware versus unaware moves. Our task used a slider puzzle game based on the well-known board game Rush Hour, and a distractor memory task consisting of background videos (Fig. 1A; see also Supplementary Video S1 in Supplemental Materials). Study activities were preapproved by the Yale University Institutional Review Board, and informed consent was obtained from all participants. Participants were instructed to remember as much detail as possible from the videos while simultaneously playing the slider game. Combining a repetitive overlearned game with an engaging distractor task was intended to encourage game play at times without awareness. Training took place on day 1 of testing and data were collected on days 2 and 3 (Fig. 1B, see Supplemental Methods for details). Periodically the game was interrupted with questions to assess awareness of the most recent game move (Fig. 1A). These included an objective multiple-choice quiz where participants were asked to choose their last move, and a subjective confidence rating that asked how certain they were of their quiz choice. Awareness was based on how certain (confident) participants were of knowing their last move, validated by correctly identifying it on the multiple-choice quiz. We found that confidence, defined by within-session percentile, was strongly related to correct responses on the quiz (Fig. 1C). Trials with high confidence (>75th percentile) had 90.8% correct block identification on the quiz (expected: 100%; Fig. 1D), whereas trials with low confidence (<25th percentile) had only 31.7% correct quiz responses (68.3% incorrect, expected: 75%; given four options at chance levels; Fig. 1D). Therefore, to robustly define AoA we defined “aware” trials as those with high confidence (>75th percentile) validated by correct quiz responses, and “unaware” trials as those with low confidence (<25th percentile) validated by incorrect quiz responses. These definitions parallel those used in perceptual awareness paradigms, where aware trials are defined as those subjectively perceived and validated by correct identification, whereas unaware trials are subjectively not perceived and validated by incorrect identification (17–20). To ensure sufficient sample sizes for analysis of electrophysiology and eye metric data, we required that all participants have a minimum of 12 aware and 12 unaware trials total across their two days of testing. Of the 77 initially enrolled participants, this yielded a total of 67 with sufficient trials for analysis (meaning that most participants exceeded the minimum number of trials needed), with an average of 32.6 ± 8.0 (mean ± SEM) aware trials and 25.5 ± 6.5 unaware trials per participant summed across the two testing days (Fig. 1E).

We investigated several demographic and task-design–related variables and found no important relationships to awareness on the task (see Supplementary Behavioral Analyses, Figs. S2 and S3, and Tables S1–S6). We also assessed the effect of delay time from action to quiz appearance on awareness rates and found that longer delay times were associated with decreased awareness rates (mean subject *r* = −0.51) and increased unawareness rates (mean subject *r* = 0.23; Fig. S4). Subsequent analyses (see below) investigated event-related potentials for short versus long delay times to ensure the observed effects were not simply due to forgetting.

### Event-related potentials: volitional and perceptual

**Figure 2:**
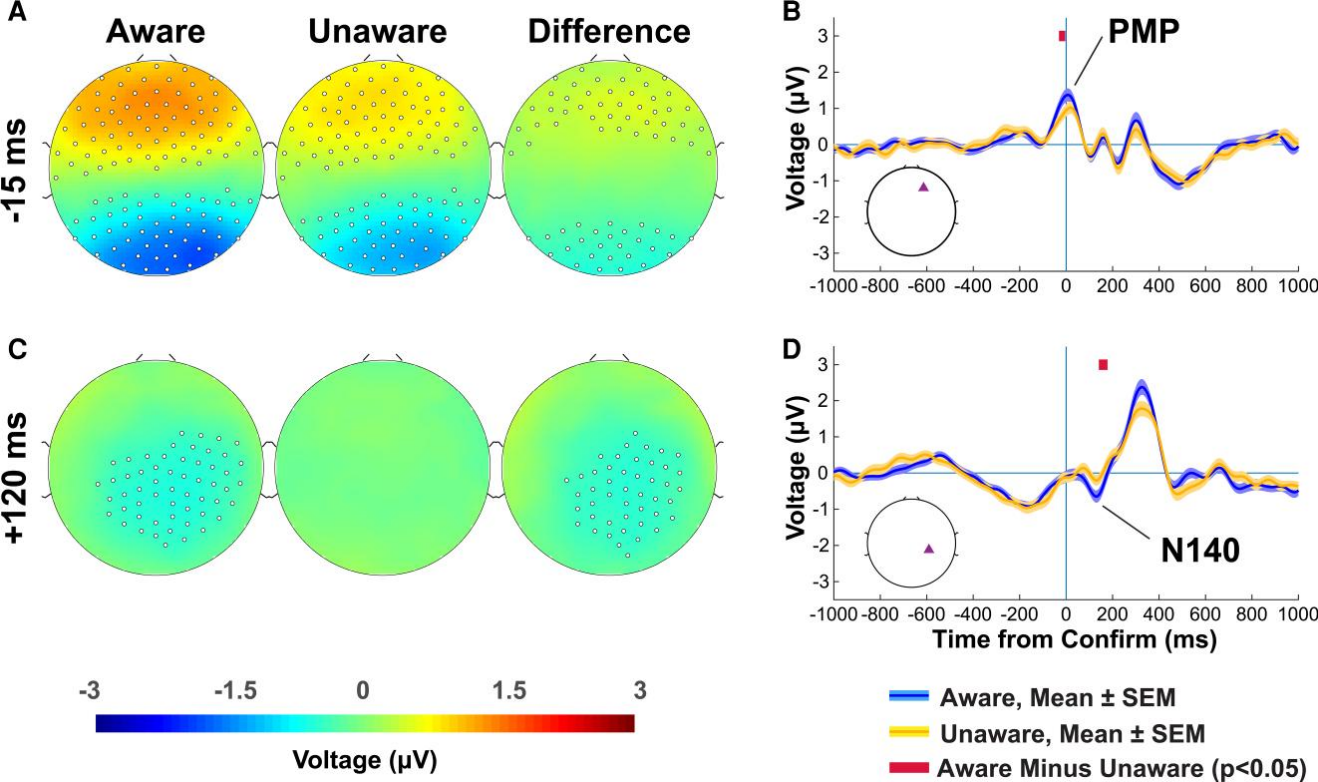
Larger volitional and perceptual event-related potentials for aware actions. Event-related potentials relative to move confirmation on the Rush Hour game for aware and unaware moves (*n* = 57 participants). A, B) Premovement positivity (PMP). Topoplots (A) show voltage at representative time points for significant clusters by spatiotemporal permutation statistics (see Supplemental Methods, *P* < 0.05) for aware, unaware, and aware minus unaware conditions, with significant electrodes indicated by black outlines. Voltage timecourse data (B) for aware and unaware trials averaged across participants (±SEM) from representative electrode within the significant PMP cluster (E5; right of Fz). Red line indicates significant (*P* < 0.05) aware minus unaware time points in the spatiotemporal cluster analysis. Time of move confirmation is *t* = 0. C, D) Postmovement somatosensory perceptual N140 event-related potential. Topoplots (C) and voltage timecourse data (E155; posterior to C4) (D) displayed with same conventions as (A, B).

To understand the preaction, efferent volitional mechanisms underlying AoA, and the postaction perceptual signals underlying AoA, we performed 256-channel high-density scalp EEG on participants as they completed the Rush Hour slider puzzle task. Immediately prior to the performance of the action, we noted a PMP (31–33) over frontal regions, which began ∼100 ms prior to action and peaked at action performance (Fig. 2A and B). The amplitude of the PMP was significantly heightened prior to aware actions when compared with unaware actions. Following the action, we noted the presence of a well-known perceptual signal, the somatosensory awareness potential, or N140 (17, 37, 38). The N140 peaked over right parietal regions (contralateral to the hand executing the action and confirmation) ∼120 ms following the action and was significantly greater in amplitude for aware actions (Fig. 2A and B). We therefore found that both volitional and perceptual neurophysiological signals were greater prior to and following aware versus unaware actions.

### Event-related potentials: precursors and consequences

**Figure 3:**
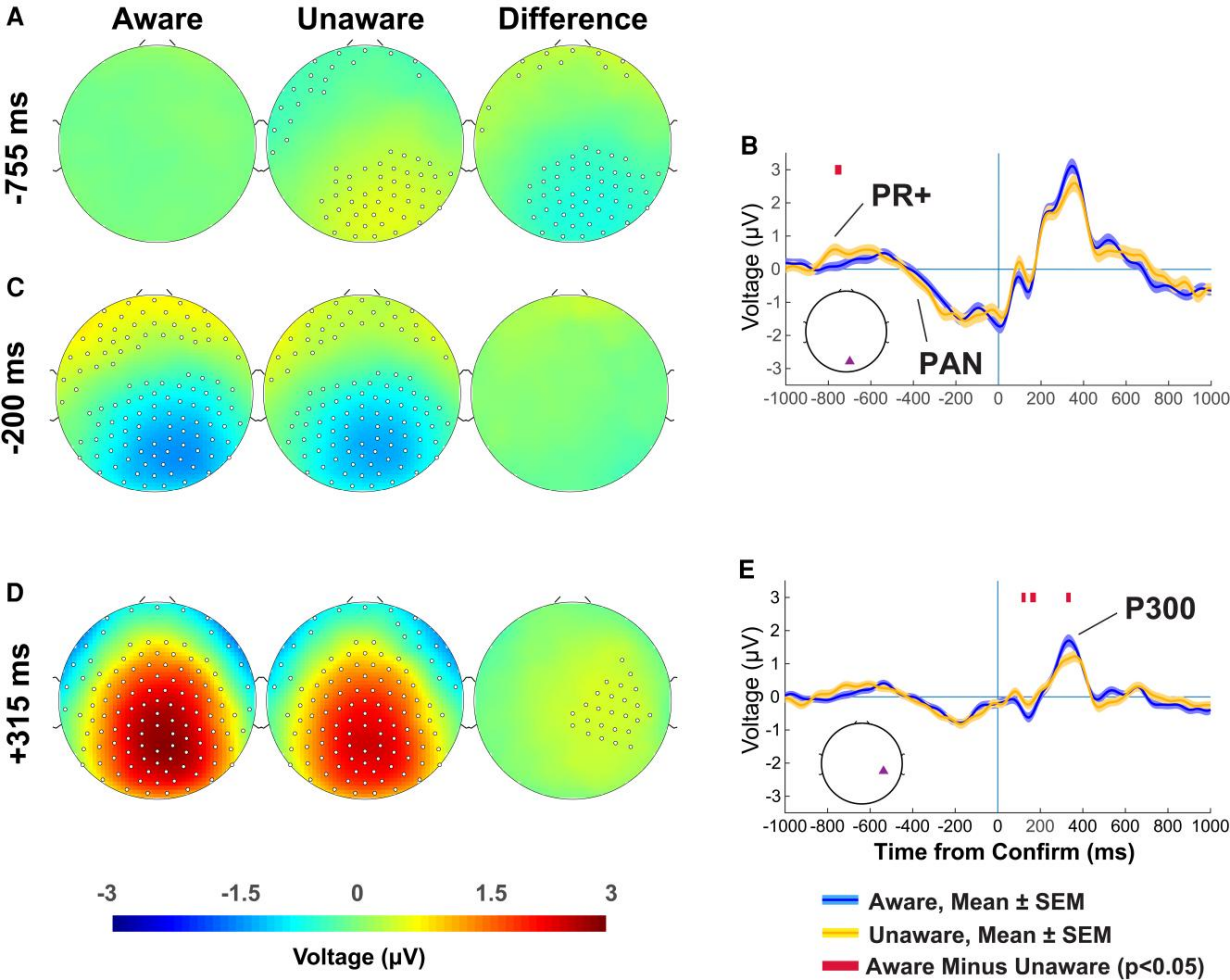
Precursors and consequences of AoA. Early and late event-related potentials relative to move confirmation on the Rush Hour game for aware and unaware moves (*n* = 57 participants). A–C). Prereadiness positivity (PR+) and preaction negativity (PAN). Topoplots show voltage at representative time points for the PR+ (A) and preaction negativity (C), with significant clusters by spatiotemporal permutation statistics (see Supplemental Methods, *P* < 0.05) for aware, unaware, and aware minus unaware conditions. Significant electrodes are indicated by black outlines. Voltage timecourse data (B) for aware and unaware trials averaged across participants (±SEM) from representative electrode within the significant PR+ cluster (E127, posterior to Pz), also showing timecourse of the preaction negativity. Red line indicates significant (*P* < 0.05) aware minus unaware time points in the spatiotemporal cluster analysis. Time of move confirmation is *t* = 0. D, E) Postperceptual P300 event-related potential (E164, posterior to C4). Topoplots (D) and voltage timecourse data (E) displayed with same conventions as (A–C). Note that in the voltage timecourses (E), in addition to significant differences for aware minus unaware signals in the positive P300, there are also significant differences during the negative N140 time range (see also Fig. 2C), due to similar posterior signal locations.

In addition to the potentials observed immediately preceding and following action, we also observed volitional precursors occurring at long timescales before (beyond 200 ms preaction) and postperceptual consequences at long timescales after (beyond 200 ms postaction) action (Fig. 3). Preaction, we observed a robust negative deflection in scalp voltage over parietal and occipital regions for both aware and unaware actions (Fig. 3C), beginning ∼600 ms prior to action and peaking ∼150 ms prior to action performance (Fig. 3B). The magnitude of this potential did not differ significantly between aware and unaware actions. This large negative premovement potential resembles the late components of the readiness potential in timing (26) but differs by being more posteriorly distributed than the late readiness potential. Prior to this negative potential, we observed a positive potential over parietal and occipital regions ∼400 to 900 ms prior to the move, which we named the prereadiness positivity (PR+; Fig. 3A and B). The magnitude of the PR+ was significantly greater for unaware actions compared with aware actions. Because the moves on the sliding block game occurred in rapid succession, we tested whether differences in the PR+ might result from carry-over from previous moves, but we did not find any significant prior move-related effects (Fig. S5).

Following perceptual attention events, late potentials such as the P300 have been related to postperceptual processing needed to subsequently report on or describe a stimulus (19, 58–62). We found a large P300 which peaked over parietal and occipital regions in a positive direction ∼350 ms following the action (Fig. 3D and E). The P300 was significantly larger following aware moves compared with unaware moves, thereby demonstrating stronger neurophysiological correlates of postperceptual processing when AoA was present.

For the event-related potential results (Figs. 2 and 3), we investigated several potential confounding factors (see Supplementary Event-Related Potential Analyses), including effects of forgetting and arousal-related criterion changes and found that any confound-related effects were not coincident with our findings (Figs. S6–S17). We also examined an alternative approach synchronizing analyses to the selection of the block (i.e. initiation of the move sequence) rather than to move completion and found no statistically significant differences between aware and unaware moves (Fig. S18). In addition, we performed bootstrap analyses to account for imbalances in aware and unaware trials within each participant or potential low sample sizes for some participants and found that our findings were robust to the bootstrapping procedure (Figs. S19 and S20).

### Power spectral analyses: alpha/beta desynchronization and frontal midline theta

**Figure 4:**
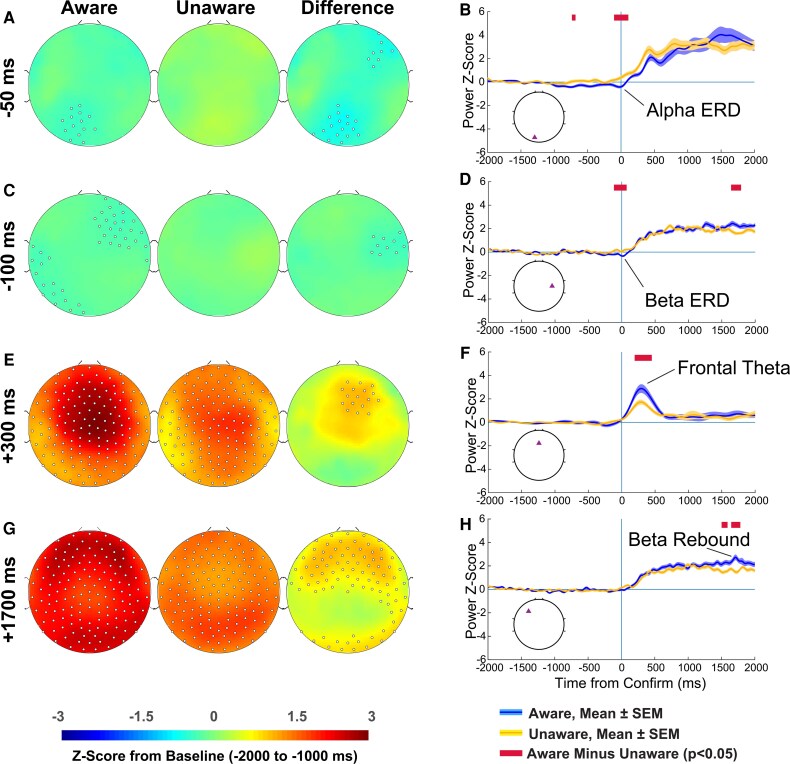
Alpha, beta, and theta frequency changes associated with AoA. Power spectral changes in frequencies of interest relative to move confirmation on the rush hour game for aware and unaware moves (*n* = 57 participants). A, B) Alpha frequency (8 to 12 Hz) event-related desynchronization (ERD). Topoplots (A) show power *Z*-scores (with −2,000 to −1,000 ms baseline for mean and SD) at representative time points for significant clusters by spatiotemporal permutation statistics (see Supplemental Methods, *P* < 0.05) for aware, unaware, and aware minus unaware conditions, with significant electrodes indicated by black outlines. Power *Z*-score timecourse data (B) for aware and unaware trials averaged across participants (±SEM) from representative electrode (E117, anterior to O1) within the significant alpha frequency event-related desynchronization cluster. Red line indicates significant (*P* < 0.05) aware minus unaware time points in the spatiotemporal cluster analysis. Time of move confirmation is *t* = 0. C, D) Beta frequency (12 to 30 Hz) ERD (E183, C4). E, F) Midline frontal theta frequency (4 to 8 Hz) changes (E15, Fz). G, H) Postmovement beta frequency rebound (E36, right of F3). Topoplots (C, E, and G) and power *Z*-score timecourse data (D, F, and H) are displayed with same conventions as (A, B).

To further understand the links between AoA and both volition and perception, we performed power spectral analyses on our EEG data focusing on previously identified event-related changes in alpha, beta, and theta frequencies (39–51, 63–67) (Fig. 4). We found that prior to aware actions, there was a significantly greater event-related desynchronization in the alpha (8–12 Hz) range over frontal and occipital regions (Fig. 4A). Alpha event-related desynchronization in aware actions began ∼1,000 ms prior to action and continued through the performance of the action (Fig. 4B). We also noted significantly greater event-related desynchronization in the beta (12–30 Hz) range over right somatomotor regions preceding aware actions (Fig. 4C). Beta event-related desynchronization in aware actions began ∼150 ms prior to action and as with alpha event-related desynchronization, continued through action performance (Fig. 4D). Following action, we noted an increase in theta power (4–8 Hz) throughout all electrodes following both aware and unaware actions; however, the magnitude of the increase in theta over midfrontal regions was greater for aware actions compared with unaware actions (Fig. 4E). The increase in theta power began immediately following action for both aware and unaware actions, and peaked ∼300 ms following aware actions and ∼250 ms following unaware actions (Fig. 4F). Lastly, we observed a robust postmovement beta rebound throughout all electrodes in both aware and unaware actions (Fig. 4G), beginning at action and reaching steady state ∼600 ms postaction. The magnitude of the postmovement beta rebound in frontal regions following aware actions significantly exceeded that of unaware actions, from ∼1,400 ms postaction onwards (Fig. 4H).

### Eye metrics: immediate

Previous work has shown transient changes in blink rate, pupil diameter, and saccade rate associated with perceptual awareness of external sensory stimuli (17, 19, 20). Therefore, to assess the relationship between AoA and these physiological markers of perceptual awareness, participants underwent pupillometry and eye tracking while performing the rush hour task. Following the confirmation of a move and subsequent disappearance of the board in preparation for a quiz, a consistent increase in blink rate was observed, beginning ∼200 ms postaction and peaking ∼500 ms postaction (Fig. S21). This increase was significantly greater in magnitude for aware actions compared with unaware actions. Postaction increases in pupil diameter (peaking ∼1,300 ms postaction) and polyphasic changes in saccade rates (peaking at ∼350 ms postaction) were observed in both aware and unaware actions and did not differ in magnitude (Figs. S21B and C).

### Behavioral and pupil physiological metrics: long term

To assess the presence of physiological metrics of fatigue or decreased arousal across runs, we analyzed preaction pupil diameter by run (Fig. S21). We calculated the average pupil diameter 1 s prior to action (−1,000 ms to 0) in all trials, regardless of awareness designation, and found consistent, statistically significant decreases in pupil diameter with each succeeding run compared with the first run. Mean pupil diameter was highest in run 1 and fell to its lowest in run 5. To evaluate the effects of fatigue and arousal on AoA, we assessed the following four key behavioral metrics across the six runs of the experiment: unawareness (percentage of unaware quizzes), awareness (percentage of aware quizzes), quiz accuracy (correct move identification), and confidence (percentile) (Fig. S21E). When compared with run 1, we found that there was a significant increase in unawareness in later runs (runs 5 and 6), a significant decrease in awareness (runs 4 and 6), a nonsignificant trend toward decreased accuracy, and a significant decrease in confidence (run 6). To determine whether AoA tended to cluster in a sustained manner, we looked at awareness levels around aware versus unaware moves (Fig. S22). The median time period between quizzes was 46.2 s, allowing us to observe the maintenance of AoA across multiple minutes. We found that if a quizzed move was aware, the three quizzed moves prior and two moves after were significantly more likely to also have been aware when compared with if the given move was unaware (Fig. S22). Awareness rates for quizzed moves even further removed in time generally did not differ based on whether the present move was aware or unaware.

## Discussion

Previous work on conscious awareness has focused primarily on neural mechanisms of sensory perception, rather than on awareness of motor action (1–5). Investigation of motor awareness has at least in part been hindered by a lack of suitable paradigms to study neural activity in aware versus unaware voluntary motor activity. To address this deficit and to resolve the long-standing Two Williams Debate, we developed a paradigm with repeated aware and unaware actions in a controlled setting. We found a wealth of neural signals differentiating aware versus unaware moves, and further revealed that neither perspective in the original debate fully captures the neural signals of AoA. Our measurements suggest that volitional as well as perceptual mechanisms both preaction and postaction play a role in AoA on short-time scales, while additional state-related arousal and attention mechanisms contribute on longer time scales. This more nuanced view of AoA is first supported by our findings that both the preaction volition-related PMP, and postaction perception-related N140 were larger for aware moves. Expanding to slightly longer timescales of volition and perception around actions, we also observed larger alpha and beta event-related desynchronization preceding aware actions, and larger frontal midline theta, P300, blink rates, and beta rebound following aware actions. Finally, on the timescale of minutes, we found—based on pupil measurements and behavior—that AoA varied with arousal state and/or fatigue during the course of the experiment. Together these results provide a broader perspective on volitional, perceptual and longer-term arousal mechanisms that may synergistically contribute to make us aware of some actions above others.

Preceding aware versus unaware moves, we observed a larger PMP, related to the immediate initiation of the action as opposed to its planning (31–33). Interestingly, at slightly earlier times we observed an EEG negativity on a similar timescale to the late components of the well-known readiness potential, thought to be related to motor intention or willful planning (15, 23–30). Our observed negativity 600 ms prior to action showed temporal but not spatial resemblance to the late readiness potential, suggesting it may be a separate action-related process, or it may differ in location because moves in our Rush Hour game occurred much more rapidly than typical readiness potential paradigms. Either way, the premove negative potential we observed did not differ between aware or unaware actions. However, at even earlier times, our preaction potential, the PR+ was significantly larger for unaware moves, suggesting that aware and unaware actions may differ in neural activity up to 800 ms preaction.

Additional evidence for combined volitional and perceptual mechanisms in AoA comes from our findings of larger alpha and beta frequency event-related desynchronization preceding aware moves, and larger postmove beta rebound following aware moves. Changes in alpha and beta frequency are well known to occur in anticipation or following volitional or perceptual events, but little work has been done previously to relate alpha or beta activity to AoA (39–51). Following aware moves, our observation of a significantly larger N140 fits with studies showing somatosensory and other negative event-related potentials in this time range related to perceptual awareness independent of task report (17, 19, 37, 38). Our findings also suggest that on longer timescales, postaction reflection plays a role in maintaining AoA and in the ability to report or describe an action. We found that the P300, a later report-dependent potential (60, 68–70), was heightened following aware actions, suggesting that AoA requires postaction attention and preparation to report. In addition to our study, the combination of the N140 and P300 has been observed across multiple somatosensory task paradigms, suggesting that the P300 is an attentional consequence of somatosensation involved in postperceptual processing (71–73). We also found increases in midfrontal theta in aware actions, a reflective mechanism seen in cognitive control tasks involving a motor component (63, 64). Our findings also provide support to other studies which have highlighted the role of midfrontal theta in successful navigation (65–67). In the longer postaction timescales (>1,500 s), we noted increases in postmovement beta rebound following aware actions. Postmovement beta rebound is generated by inhibition of motor cortex and preparation for sensory feedback of action, a long-term consequence of action (74–77).

Eye metrics have been shown previously to provide insights into perceptual awareness, with transient changes in blink rate, pupil diameter, and saccade rate occurring following perception of sensory stimuli regardless of modality including vision, hearing, and touch (17, 19, 20, 78–80). Specifically, previous work in humans (17, 19, 20) and nonhuman primates (81) has shown a transient increase in blink rate after consciously perceived sensory stimuli. The eye metrics in our findings show significantly greater increases in spontaneous blink rate following aware actions, providing additional evidence that perceptual mechanisms contribute to AoA. The lack of significant effects of AoA on transient pupil diameter or saccades might in part be related to our task paradigm, where the move confirmation coincided with disappearance of the game screen, so that the associated changes in luminance or fixation might have obscured any relevant transient pupil or saccade-related signals. Therefore, these eye metric signals should be investigated in future work using modified paradigms without these potential confounds.

We found behavioral patterns and physiological measurements suggesting a role of arousal and fatigue in the maintenance of AoA on longer time scales. These patterns and biological signals occurred over the course of multiple seconds or even over the course of minutes. Our sequential quiz-to-quiz analyses (Fig. S22) suggest that periods of awareness are maintained for ∼4.5 min, nearly half of an entire run. Our run-by-run behavioral findings of decreased confidence, accuracy, and awareness, as well as increased unawareness rates over time, suggest that long-term decreases in arousal or fatigue compromise AoA. Our run-by-run decreases in pupil diameter support our behavioral findings, providing physiological evidence for decreased arousal across runs. Given that previous work has observed that criterion placement can impact effect sizes (82), and observing fatigue-related changes in confidence designation, we assessed whether our N140, PMP, P300, and novel PR+ findings might be impacted. We found that while certain event-related potentials changed between the earlier and later phases of the experiment, these changes, frontally, did not coincide with the parietal location of our aware and unaware differences. Ultimately of course subjective criterion-related concerns for any report-based paradigm are best addressed by the use of no-report paradigms for evaluating awareness, which should be developed for studying AoA in future studies (see for example (19) for one potential approach).

As in most paradigms of perceptual awareness, our paradigm for AoA evaluated awareness retrospectively, with a delay between the events being studied and subsequent report. The advantage of introducing a brief delay between events and report is that this allows neural signals to be observed at the time of the aware event itself and prior to signal confounds from the report probe. However, the delay also introduces a limitation because awareness is not assessed simultaneously with its occurrence. For example, in our paradigm the time between action and quiz of 2 to 8 s (Fig. 1A) raises the possibility that some trials with reported loss of awareness were in fact briefly aware but were later reported as unaware due to forgetting. This question is mitigated at least in part by several considerations as we will now discuss in more detail. First of all, it is certain that given sufficient time, such as the course of minutes or hours, the ability to report a move or to report any event will diminish (83). A similar question could be asked of any paradigm where there is a delay in report of awareness, including many studies of perceptual conscious awareness that rely on delayed report. Although is it clear that ability to report an event will decline over time, we observed differences in physiological metrics well before and within the bounds of iconic memory (a few hundred milliseconds) (84, 85). The observed physiological differences coinciding with, immediately after and even preceding aware versus unaware actions on such short-time scales are more likely related to awareness at the time of the action than to subsequent forgetting rates. Furthermore, the time delay between the action performed and the presentation of a quiz (2 to 8 s) remains within the bounds of short-term memory, measured on the scale of tens of seconds (86, 87). It is therefore unlikely that unaware actions on such short-time scales are purely due to forgetting or memory-related mechanisms. Nevertheless, to ensure that the physiological signals we observed were not affected by forgetting, we compared EEG results for short versus long delays and found no significant differences (see Supplementary Event-Related Potential Analyses, Figs. S6–S9). Interestingly, we did observe a decrease in awareness and an increase in unawareness with longer delays. However, the lack of EEG differences for short versus long delays suggests that even if some forgetting occurs in our paradigm, this effect is not sufficient to change the main EEG signals for aware and unaware moves. Future work should investigate in greater detail whether or not AoA may be more susceptible to forgetting than perceptual awareness paradigms with similar time delays (17–20). Finally, it should be acknowledged that the current experiments cannot fully exclude an alternative explanation of the results based on rapid forgetting. It is theoretically possible that some moves are initially aware or potentially aware but then are very rapidly forgotten even by the time of the earliest question time 2 s after the move. This could be tested in future studies by probing awareness with no delay or by rapidly redirecting attention to the relevant portion of the testing screen to evaluate the full capacity for spatial awareness, as has been done for perceptual awareness paradigms (84, 88, 89). Extrapolating back from the gradual decrease in reported awareness that we observed over time (Fig. S4) argues against an abrupt increase in the proportion of aware events at times earlier than 2 s, but this possibility should nevertheless be investigated in future work.

While our study has encapsulated much of the action patterns and planning seen in naturalistic AoA, it still remains to be seen if the kinesthetics underlying an action affect AoA. Our study has probed navigation, only one naturalistic situation under which AoA is lost. Other effortful actions, such as those involving physical fatigue, or grasping while obstructed may show diverging neural pathways (90–92). Furthermore, a whole body experience may differ from a simple point-and-click game, especially when coordinating multiple limbs (93, 94). Additionally, our study has also probed only the cortical mechanisms of AoA. Given that action is known to be generated in the cerebellum, brainstem, basal ganglia, and other subcortical regions, further studies with fMRI and other imaging modalities are needed (95–98). Although we found no significant relationship between awareness and overall familiarity or engagement with the background video (Tables S4 and S5), future work should examine whether moment-to-moment fluctuations in video content with high or low attentional salience might influence AoA. Lastly, our behavioral experiment is readily adaptable to interventional modalities which have been tested on motor regions, such as transcranial magnetic stimulation and transcranial alternating current stimulation (99–102). These studies will allow causal neuroanatomical determinants of AoA to be conclusively identified.

The overlap between findings in our clinically normal population and populations with impaired AoA is notable. Two of our time-frequency findings, midfrontal theta and beta desynchronization, are known to be diminished in patients with Parkinson's disease (103–105). Furthermore, premovement event-related desynchronization and postmovement beta rebound are attenuated in amylotrophic lateral sclerosis, and beta hypersynchronization is noted in dystonia (104, 106). This suggests that the aberrant AoA seen in these neurological disorders may be related to loss of control over the normal awareness processes. Further development of behavioral paradigms to explicitly evaluate AoA in clinical populations will be needed to test this hypothesis, and to determine the potential value of AoA assessment in diagnostic batteries.

Our results provide a definitive answer to the Two Williams Debate. Given our findings of both preaction and postaction differences in neural activity between aware and unaware actions, we conclude that it is impossible to ascribe the neural bases of AoA to purely a priori, volitional or purely a posteriori, perceptual mechanisms. Thus, both perspectives in the Two Williams Debate hold merit in explaining the mechanisms underlying action. Furthermore, our longer-term findings in the precursors and consequences of action suggest that the dichotomous view of action mechanisms as either preaction or postaction is incomplete. These long-term findings demonstrate that beyond change in dynamic, transient neural activity, AoA is also characterized by static, long-term states. With these in findings mind, we can summarize our results in the context of a wider framework of volition and perception to provide a broad conceptual model of AoA (Fig. S23 and Table S6). We hypothesize first that AoA occurs in the context of long-term modulation of arousal and attention states which bias the volitional and perceptual mechanisms acting on shorter time scales. Next, prior to voluntary movement, intention and planning may lead to early differences in brain physiology between aware and unaware actions, such as the PR+. The subsequent volitional and perceptual mechanisms include movement initiation and performance, as well as several perceptual stages. We recently summarized the perceptual side of the story with a Detect, Pulse, Switch, and Wave model (107), which we expand here, adding movement initiation/performance to obtain a comprehensive framework for AoA (Fig. S23 and Table S6). On the side of volition, our findings have provided concrete evidence for signals related to intention/planning (PR+), action initiation and performance (PMP, alpha and beta event-related desynchronization). On the side of perception, other studies show evidence for the detect, pulse and switch mechanisms, not studied in the present investigation (18, 19, 107–109). However, the present work does provide evidence for perceptual and postperceptual mechanisms in AoA by showing that the N140, P300, midfrontal theta, and postmovement beta rebound are all larger for aware actions. Further investigation of AoA is needed to more fully establish the roles individual volitional and perceptual mechanisms and of longer-term arousal states as necessary and sufficient for AoA. Through the perspective of individual events and overarching states, this and future work may lead to a deeper understanding of how the combination of multiple systems in tandem builds the richness of the human experience of AoA.

## Supplementary Material

pgag220_Supplementary_Data

## Data Availability

All anonymized data created for the study are available in the Yale Dataverse: https://dataverse.yale.edu/dataverse/aoa/. All code used in this project are in the following GitHub repository: https://github.com/BlumenfeldLab/Jin-et-al_2026/.

## Preprints

An earlier version of this manuscript was posted as a preprint at https://www.biorxiv.org/content/10.1101/2024.08.15.608153v1.
