# Supplementary material for: Neural mechanisms of awareness of action
Source: PNAS Nexus. 2026 Jul 2;5(7):pgag220. doi: 10.1093/pnasnexus/pgag220 (PMC13340929; doi:10.1093/pnasnexus/pgag220)
Supplement: pgag220_Supplementary_Data [file pgag220_supplementary_data.zip › Jin 2026 AoA EEG Eyes and Behav-26 Supplement.pdf]

Supplementary Material for:

Neural mechanisms of awareness of action

David S. Jin[1,4], Oumayma Agdali[1,4], Taruna Yadav[1], Sharif I. Kronemer[1,4], Sydney Kunkler[1], Shweta Majumder[1], Maya Khurana[1], Marie McCusker[1,4], Ivory Fu[1], Emily J. Siff[1,4], Aya Khalaf[1], Kate L. Christison-Lagay[1], Shanae L. Aerts[1,4], Qilong Xin[1,4], Jing-Jing Li[1], Sarah H. McGill[1,4], Michael J. Crowley[5], and Hal Blumenfeld[1,2,3,4]

Departments of [1]Neurology, [2]Neuroscience, [3]Neurosurgery, Yale University School of Medicine, 333 Cedar Street, New Haven, Connecticut 06520, USA

[4]Interdepartmental Neuroscience Program, Yale University

Hope 212, P.O. Box 208074, New Haven, CT 06520, USA

[5]Child Study Center, Yale University School of Medicine

230 S. Frontage Road, New Haven, CT 06519, USA

Supplementary Results

Supplementary Behavioral Analyses

We found no relationship between block identity (red block vs. white block) on awareness/unawareness outcomes (**Supplementary Table S1**). We found no effects of sex on awareness, unawareness, raw confidence, and accuracy (**Supplementary Table S2**). We found no effects of age on awareness, raw confidence level, and accuracy, and a statistically significant, albeit small relationship between age and unawareness rates ($r = -0.29$, $p = 0.02$; **Supplementary Fig S2**). Of our 67 participants, 61 completed both testing days of the experiment. We found no effects of testing day on unawareness, awareness, accuracy, nor confidence. (**Supplementary Table S3**). We found no relationship between awareness outcomes and the difficulty of a puzzle in which an action was performed (measured by minimum number of moves needed to solve the puzzle; **Supplementary Fig S3**). In addition, we observed no statistically significant difference in total move time (from block selection to move confirmation) for aware (469 ± 26ms; median ± standard error of the median) versus unaware (458 ± 25 ms) actions ($N = 57$, $p = 0.19$).

To assess the effects of the background distractor task on accuracy and awareness rates, we had participants rate each video on a 5-point Likert scale for both their familiarity with the video and its subject matter, and their interest and engagement with the video. The familiarity quiz was completed by 45 participants and the engagement quiz was completed by 46 participants. We found no effects of familiarity or engagement on awareness, unawareness, accuracy, nor confidence, neither within participants nor across participants (**Supplementary Tables S4, S5**).

Supplementary Event-Related Potential Analyses

As mentioned in the paper main results section, we found that longer delay times from action to quiz appearance were associated with decreased awareness rates and increased unawareness rates (**Supplementary Fig S4**). Therefore, we compared event-related potential findings (Figures 2 and 3) for short versus long delay times to ensure the observed effects were not simply due to forgetting. We compared short-delay (2-5s) aware actions with long-delay (6-8s) aware actions, and found no difference in the main signals (PMP, N140, PM+, P300) described in Figures 2-3 (**Supplementary Fig S6-S7**). We also performed the same analysis, comparing short-delay unaware actions with long-delay unaware actions, and again found no difference in the main signals described in Figures 2-3 (**Supplementary Fig S8-S9**).

To assess the impact of arousal-related changes in confidence and accuracy on event-related potentials, we compared aware actions of early runs (runs 1-3) against aware actions of late runs (runs 4-6). We found no differences in the signals described in **Fig 2** (PMP, N140; **Supplementary Fig S10**). We observed a heightened P300 over frontal electrodes in early runs compared to late runs (**Supplementary Fig S11**). As the difference between aware and unaware P300 exists over right parietal and occipital electrodes, these findings are not spatially coincident with our findings between aware and unaware actions. We also compared early run unaware actions against late run unaware actions. At the corresponding timepoint to the N140, we found a slight frontal positivity in early-run unaware actions compared to late-run unaware actions (**Supplementary Fig S12**). As our observed N140 difference in aware and unaware actions occurred parietally, these findings are also not spatially coincident with our previous findings. We found no differences in the early run unaware versus late run unaware signals described in

Fig 3 (PR+, N140; **Supplementary Fig S13**).

As in previous studies of perceptual awareness [1-4], we defined aware trials as those with subjective high confidence of awareness and objective correct identification, and unaware trials as those with subjective low confidence of awareness and objective incorrect identification. The advantage of this approach is that both awareness and unawareness are validated by objective testing. In other words, this approach ensures that aware trials are those in which participants subjectively report awareness (high confidence) and are reliably performing the task by correct identification, thereby eliminating "false positive" high confidence trials with incorrect identification. Similarly, unaware trials are those in which participants subjectively report unawareness (low confidence) and have incorrect objective identification, thereby eliminating "blindsight" or "lucky guess" low confidence trials with correct identification. Although studying neural signals associated with false positive perception or blindsight are valuable goals in themselves, as in previous studies of perceptual awareness [1-4], our awareness of action paradigm was explicitly designed to minimize errors due to high confidence trials with incorrect identification or low confidence trials with correct identification (Figure 1 C, D). For example, with an average of 32.6 aware trials per participant and a 9.4% error rate, there were an average of only 3.4 high confidence error trials per participant. However, a disadvantage of this approach in defining awareness based on a combination of confidence and accuracy, is that it does not easily allow analysis of confidence alone or accuracy alone. Therefore, to assess overall effects of accuracy within the high- and low-confidence trials, we compared ERP signals of correct trials and incorrect trials aggregated from the top and bottom quartiles only. Similarly, to

separately assess the overall effects of confidence, we also performed a comparison of all high- and low-confidence trials, irrespective of accuracy.

To assess whether the difference between aware and unaware actions can be explained by accuracy alone, we compared all correctly identified actions in the top and bottom quartiles (regardless of whether or not they were validated with high confidence) against all incorrectly identified actions in the top and bottom quartiles (regardless of whether or not they were validated with low confidence). When comparing ERP signals of correct vs incorrect trials, we observed similar trends to aware and unaware trials, but no statistically significant differences other than a small region of the PMP. Thus, we observed statistically significant differences in the topoplots for the PMP, albeit to a lesser spatial extent than the original aware vs. unaware comparison (**Supplementary Fig S14**). We observed no statistically significant differences in the N140, albeit a similar trend to our main results (**Figure 2**). We observed no statistically significant differences in the PR+ nor P300, albeit again a similar trend to our main results (**Supplementary Fig S15**).

To assess whether the difference between aware and unaware actions can be explained by confidence alone, we compared all high-confidence actions against all low-confidence actions. When comparing ERP signals of high-confidence and low-confidence trials, we observe a statistically significant PR+, and a smaller number of statistically significant electrodes in the PMP and P300. Thus, we observed statistically significant differences in the PMP, but to a lesser spatial extent compared to the aware vs. unaware comparison in the main results (**Supplementary Fig S16**). We observed no statistically significant differences in the N140,

albeit a similar trend to the main results (**Figure 2**). We observed a PR+ similar in extent to our original aware vs. unaware comparison (**Figure 3**) and statistically significant differences in the P300, albeit to a lesser spatial extent (**Supplementary Fig S17**).

The above findings suggest that awareness of action cannot be explained by a single factor of accuracy or confidence alone. Although both task accuracy and confidence each contribute to signals trending towards those seen in our main results, it is only by combining these factors in the definition of awareness as used in the present paradigm, which enables the full physiological signals to be seen.

Although all analyses in the present study were aligned to the final move completion or confirmation, the "action" in reality was a sequence of actions that took a total of less than ~500ms to complete on average. As described in the Supplementary Methods, our action sequence consisted of the following three parts; 1. Selection of the block via a mouse click, 2. Use of the WASD keys to move the block, and 3. Confirmation (completion) of the action by spacebar press. The first and third components of the action sequence were temporally logged via TTL pulses. Due to the rapidity of the individual movements, a TTL pulse for the WASD keypresses could not be logged for technical reasons. However, as an alternative approach instead of synchronizing our analyses to the final move confirmation, we repeated our event-related potential analyses synchronized to the initial block selection (**Supplementary Fig S18**). This showed several event-related potentials that can be interpreted as blurred versions of potentials related to the move sequence and its completion, but interestingly, did not demonstrate significant differences between aware and unaware moves. Prior to the initial block selection we

observed a slow-moving positivity, perhaps representing an extended version of the premovement positivity (**Supplementary Fig S18A; Fig 2A**). Next, there was a gradual negative deflection. Given that the final move confirmation would occur ~400-500ms on average after the initial block selection click, the timing and distribution of this potential suggest it represent a pre-action negativity for the final move confirmation (**Supplementary Fig S18B; Fig 3C**). Finally, we observed a positivity, spatio-temporally coincident with a blurred version of the P300 associated with the final move confirmation (**Supplementary Fig S18C; Fig 3D**). In summary, reanalyzing the results temporally aligned to the beginning (block selection) rather than the end (move confirmation) of the action sequence let to less coherent event-related potentials with no significant differences between aware versus unaware moves, supporting our choice of final move confirmation as the time point of interest for analysis.

Supplementary Table S1 Relationship between key behavioral metrics and block color

	Red[1] N = 56[2]	*White*[1] N = 56[2]	*p-value*[3]
Awareness[4]	20.9±3.0%	21.2±1.0%	0.84
Unawareness[4]	20.7±3.2%	17.8±1.1%	0.34
Accuracy[4]	64.3±5.1%	65.3±4.2%	0.84
Confidence[4]	49.2±2.1%	48.8±0.5%	0.84

[1]Red blocks were the target block which must be moved to solve the puzzle, while white blocks were any other obstructing block (**Fig 1A**).
[2]A total of 67 participants were included, but block identity (red versus white) was not saved for the first 11 participants for technical reasons, resulting in sample size of 56 for this analysis.
[3]Paired two-tailed t-test with Benjamini-Hochberg FDR correction for multiple comparisons.
[4]See Methods for definitions. Values shown are mean across participants ± 95% confidence intervals

Supplementary Table S2 Relationship between key behavioral metrics and sex

	Male<br>N = 24	*Female*<br>N = 43	*p-value*[1]
Awareness[2]	21.3±1.7%	21.3±1.0%	0.99
Unawareness[2]	17.6±1.9%	18.4±1.3%	0.61
Accuracy[2]	67.6±7.2%	64.4±4.6%	0.61
Confidence[2]	59.1±5.9%	54.9±4.6%	0.61

[1]Two-tailed two-sample t-test, with Benjamini-Hochberg FDR correction for multiple comparisons.
[2] See Methods for definitions. Values shown are mean across participants ± 95% confidence intervals. Values shown are mean across participants ± 95% confidence intervals.

Supplementary Table S3 Relationship between key behavioral metrics and testing day

	Day 1 *N=61*[1]	*Day 2* *N=61*[1]	*p-value*[2]
Awareness[3]	21.7±1.1%	22.3±1.1%	0.50
Unawareness[3]	17.4±1.0%	17.0±1.2%	0.61
Accuracy[3]	64.9±4.1%	67.6±4.5%	0.15
Confidence[3]	57.0±3.7%	57.9±4.0%	0.98

[1]A total of 67 participants were included, but only 61 had data on both testing days.
[2]Two-tailed paired t-test, with Benjamini-Hochberg FDR correction for multiple comparisons.
[3]See Methods for definitions. Values shown are mean across participants ± 95% confidence intervals.

Supplementary Table S4 Relationship between key behavioral metrics and video engagement

	Average ρ[1]	*N Significant Positive ρ*[2]	*N Significant Negative ρ* [2]	*N Non-Significant ρ*[2]
Awareness[3]	-0.01	1	2	43
Unawareness[3]	0.06	2	0	44
Accuracy[3]	0.03	1	2	43
Confidence[3]	-0.04	1	1	44

[1]Spearman correlation coefficient ρ was calculated to relate video engagement questionnaire scores to the four key behavioral metrics across runs within each participant. Average correlations between video engagement and all four behavioral metrics were low across participants.
[2]Spearman correlation coefficient ρ in each participant was followed by t-test for significance ($p<0.05$). Very few participants (N) had significantly positive or negative correlations between video engagement and any behavioral metrics. Total sample size was 46 participants who completed the engagement questionnaire.
[3]See Methods for definitions.

Supplementary Table S5 Relationship between key behavioral metrics and video familiarity

	Average ρ[1]	*N Significant Positive ρ*[2]	*N Significant Negative ρ*[2]	*N Non-Significant ρ*[2]
Awareness[3]	0.02	2	2	41
Unawareness[3]	0.08	2	0	43
Accuracy[3]	0.00	1	1	43
Confidence[3]	0.00	1	2	42

[1]Spearman correlation coefficient ρ was calculated to relate video familiarity questionnaire scores to the four key behavioral metrics across runs within each participant. Average correlations between video familiarity and all four behavioral metrics were low across participants.
[2]Spearman correlation coefficient ρ in each participant was followed by t-test for significance ($p<0.05$). Very few participants (N) had significantly positive or negative correlations between video familiarity and any behavioral metrics. Total sample size was 46 participants who completed the engagement questionnaire.
[3]See Methods for definitions.

Supplementary Table S6 Model of Awareness of Action – Contributing Mechanisms and Metrics

Volition		Perception	
**Mechanisms**	**Metrics**	**Mechanisms**	**Metrics**
Intention/Planning	PR+	Detect, Pulse, Switch	FEF increase, TAP, DMN decrease
Initiation	PMP, Alpha-Beta ERD	Early awareness signals	N140, (VAN, AAN)
Performance	PMP, Alpha-Beta ERD	Post-perceptual processing	P300, PMBR, Midfrontal Theta

PR+, pre-readiness positivity; PMP, pre-movement positivity; ERD, event-related desynchronization; FEF, frontal eye fields; TAP, thalamic awareness potential; DMN, default-mode network; N140, early somatosensory perception-related event-related potential; VAN, visual awareness negativity; AAN, auditory awareness negativity; P300, late post-perceptual processing-related event-related potential; PMBR, post-movement beta-rebound. See also **Supplementary Fig S23**.

Supplementary Figure S1

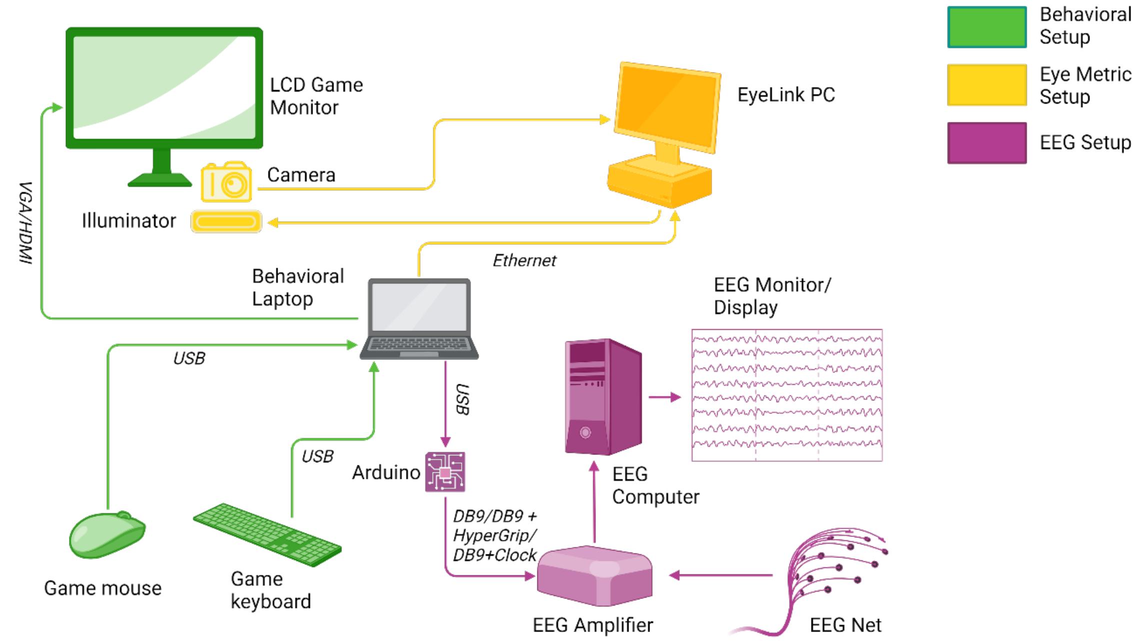

Supplementary Figure S1 Experimental setup. Participants performed the behavioral task using items shown in green. Eye metric-related items are shown in yellow, and EEG-related items are shown in purple. The behavioral laptop to EEG amplifier connection was established via Arduino with three methods (1) direct DB9 connection (N = 31 participants), (2) DB9 to HyperGrip (N=28 participants), and (3) DB9 to clock box (N = 8 participants). *Created with BioRender.com.*

Supplementary Figure S2

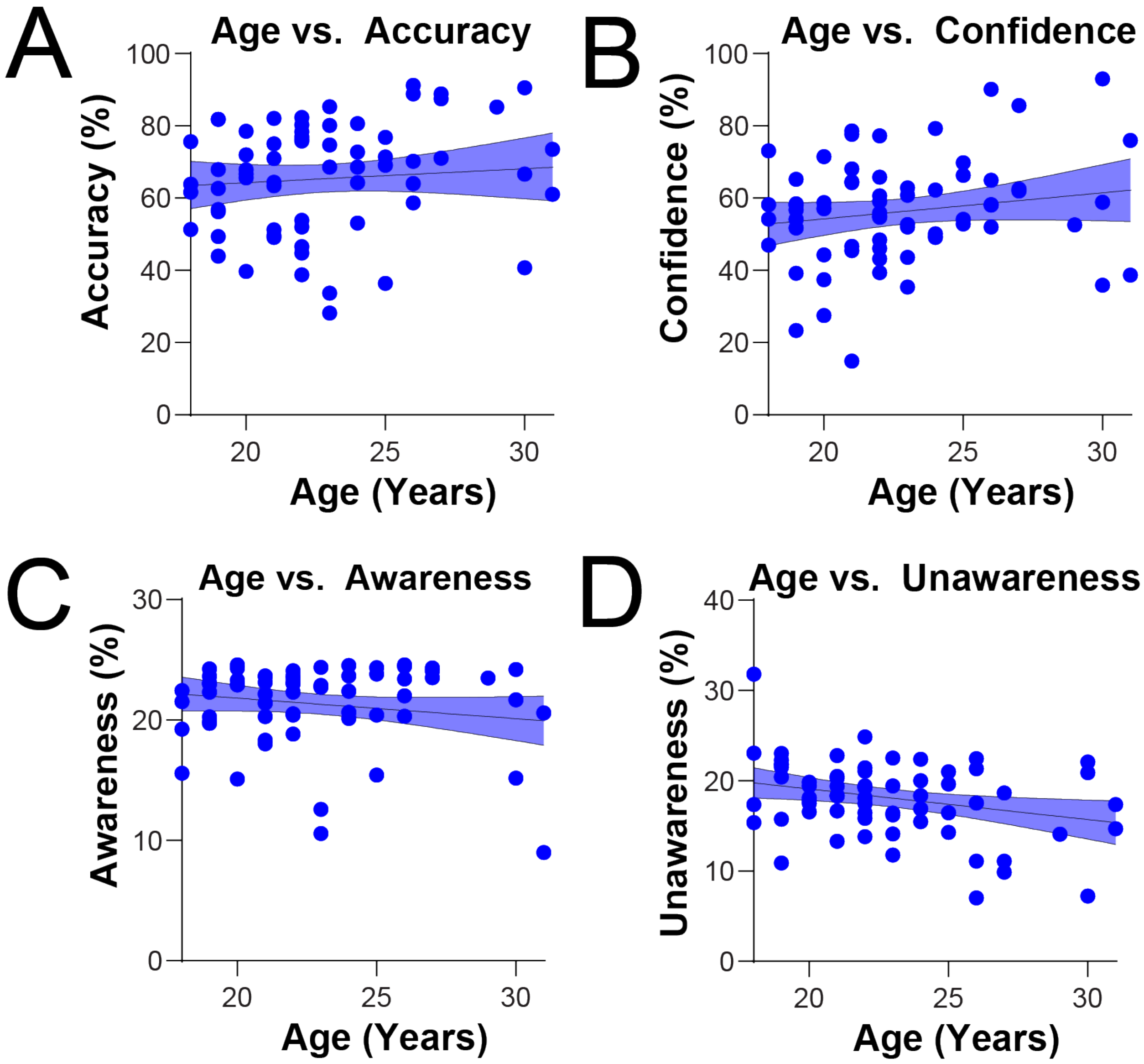

Supplementary Figure S2 Relationship between age of participant (N = 67) age and key behavioral metrics of **A.** Accuracy (ρ = 0.09, p = 0.47), **B.** Confidence (ρ = 0.18, p = 0.15), **C.** Awareness (ρ = -0.18, p = 0.15) and **D.** Unawareness (ρ = -0.29, p = 0.02*). Spearman correlation coefficient followed by t-test for significance (P <0.05, *).

Supplementary Figure S3

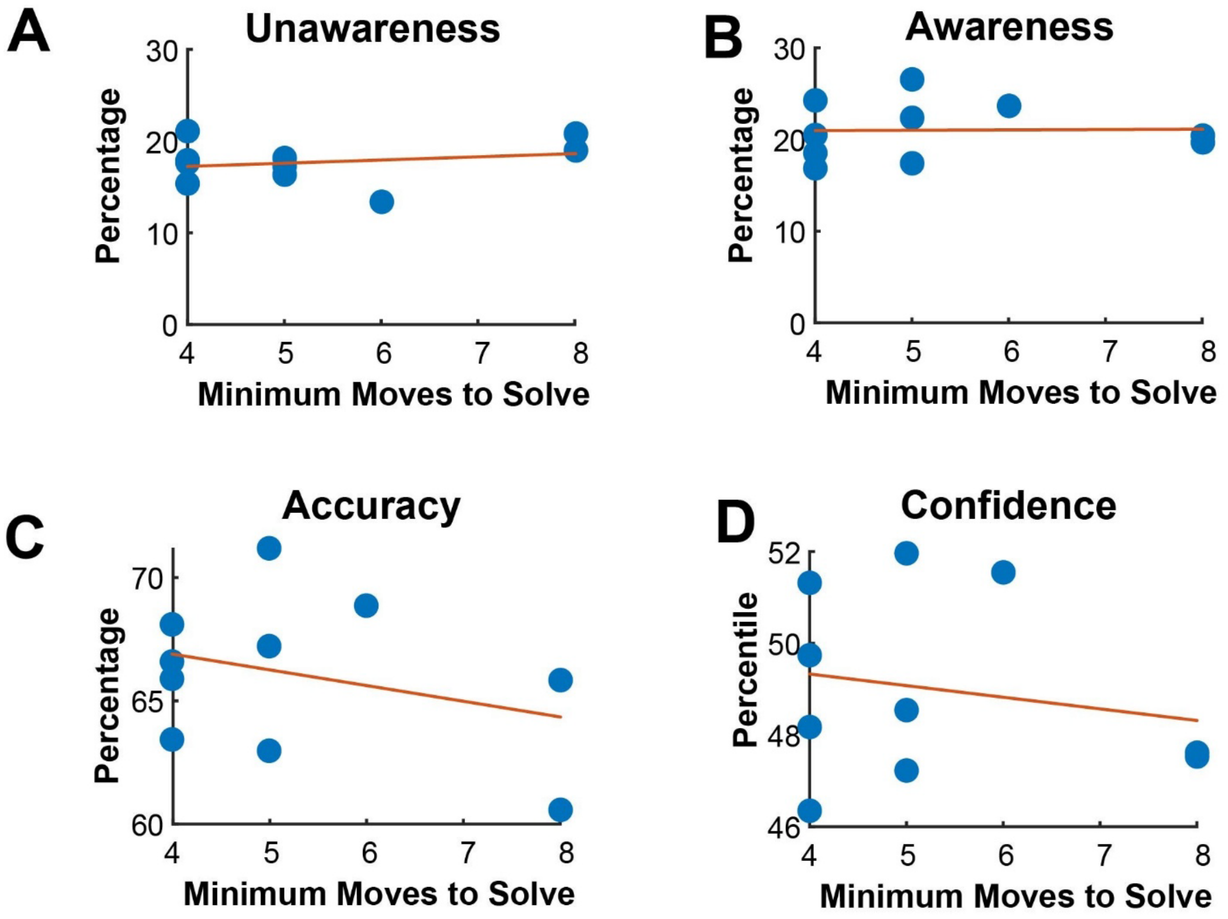

Supplementary Figure S3. Relationship between awareness metrics and puzzle difficultly, represented by the number of moves needed at minimum to solve the puzzle (N = 56 participants, n = 8,406 quizzes). A. Unawareness (r = 0.23, p = 0.52), B. Awareness (r = 0.02, p = 0.96) C. Accuracy (r = -0.32, p = 0.36), and D. Confidence (r = -0.20, p = 0.58). Each point represents an individual starting puzzle configuration, amongst the 10 total unique individual puzzle configurations. A total of 67 participants were included, but block identity (and thus puzzle configuration) was not saved for the first 11 participants for technical reasons, resulting in sample size of 56 for this analysis.

Supplementary Figure S4

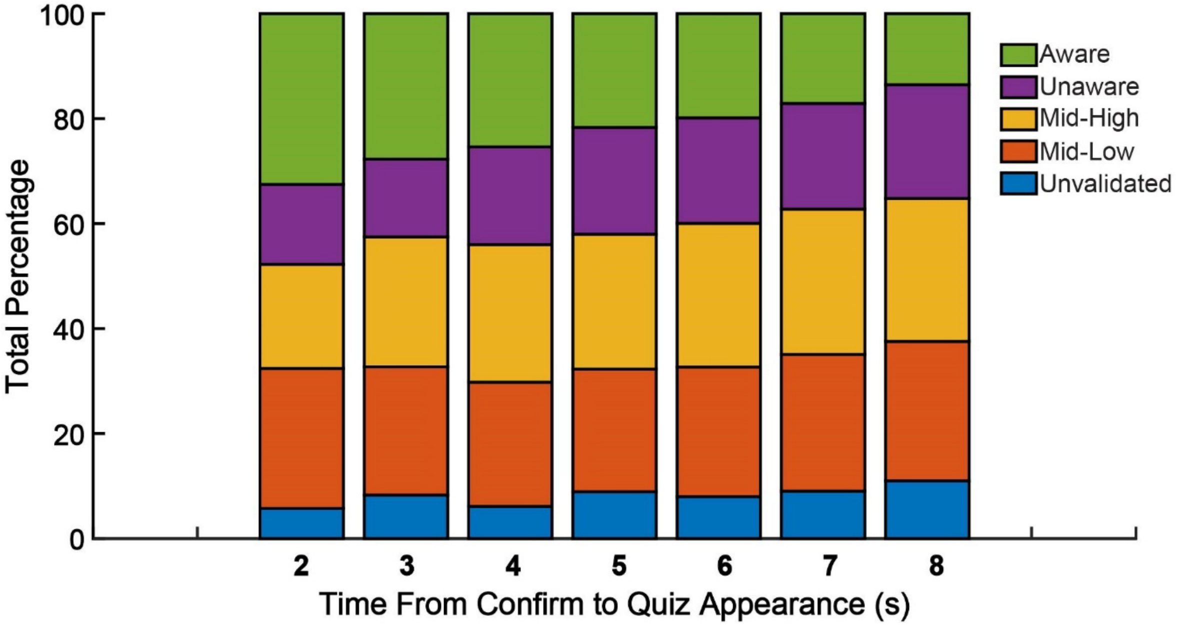

Supplementary Figure S4. Relationship between delay (time from the confirmation of the action to the appearance of a quiz) and awareness, unawareness, and other trial types (N = 53 participants). Subject awareness rates (green) decreased (mean subject r = -0.51) and unawareness rates (purple) increased (mean subject r = 0.23) with a longer delay between action and quiz. For completeness, other trial types are also shown (see Figure 1C, D) including mid-high confidence trials (yellow, 50 to 75% confidence percentile, regardless of accuracy), mid-low confidence trials (orange, 25 to 50% confidence percentile, regardless of accuracy), and unvalidated trials (blue, high confidence trials (75 to 100%) with incorrect quiz identification and low confidence trials (0 to 25%) with correct quiz identification). A total of 57 participants were included in the analysis, but due to technical reasons, delay data were not saved for four, resulting in a final total of 53 participants.

Supplementary Figure S5

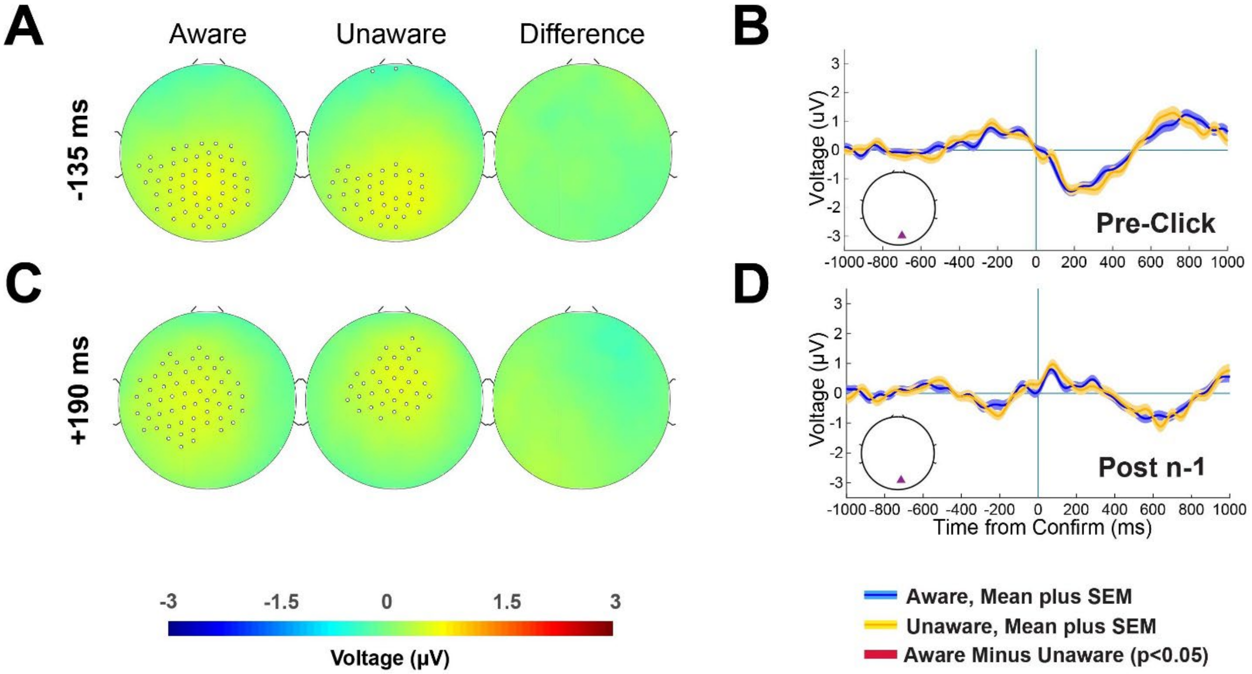

Supplementary Figure S5 Assessment of preceding actions for potential leakage to the PR+. Two salient actions occurring immediately prior to the move confirmation space bar press are 1. the block selection mouse click (Pre-Click), and 2. the move confirmation press for the preceding move (Post n-1) (see Methods: Task Design; **Fig 1A** and **Supplementary Video S1**). On average the Pre-Click occurred ~400 ms prior to move confirmation, and the Post n-1 move confirmation occurred ~1,000 ms prior to the present move confirmation. We were interested in possible leakage of these actions into the time period ~400 to 900 ms prior move confirmation where the PR+ is seen (**Fig 3B)**. These correspond to times from ~500 ms to 0 ms prior to the Pre-Click, and times ~100 ms to 600 ms after the Post n-1 move confirmation. Therefore, we analyzed event-related potentials centered on the Pre-Click time or centered on the Post n-1 move confirmation time. Aware and unaware trials were defined based on the present move (N=57 participants). **A, B.** Event-related potentials relative to the Pre-Click. Topoplots (**A**) show voltage at representative time points for significant clusters by spatiotemporal permutation statistics (see Methods, $p<0.05$) for aware, unaware, and aware minus unaware conditions, with significant electrodes indicated by black outlines. Voltage timecourse data (**B**) for aware and unaware trials averaged across participants (±SEM) from representative electrode within the significant PR+ cluster (See **Fig 3 A, B**). No significant ($p<0.05$) aware minus unaware time points were found. Time of Pre-Click is t=0. **C, D.** Event-related potentials relative to the Post n-1 move confirmation. Topoplots (**C**) and voltage timecourse data (**D**) displayed with same conventions as (**A, B**).

Supplementary Figure S6

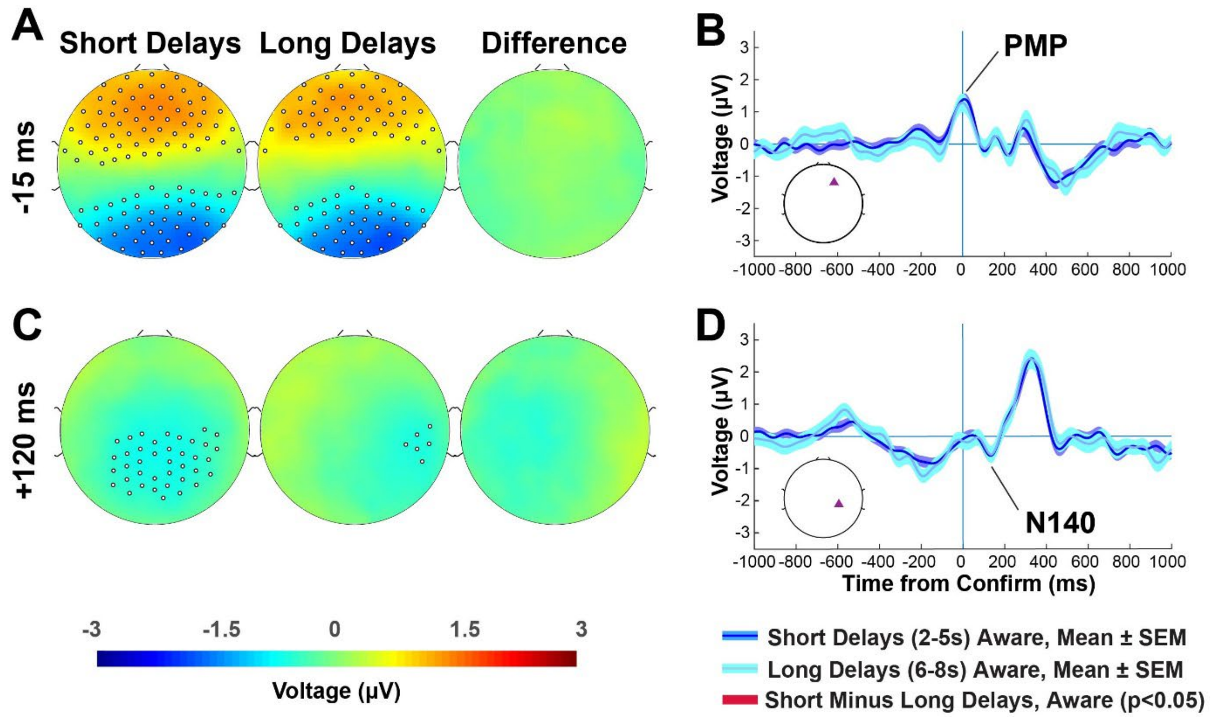

Supplementary Figure S6 (Related to Figure 2). Comparison of event-related potentials for aware actions, short delays vs. long delays. Event-related potentials relative to move confirmation on the Rush Hour game for short-delay aware and long-delay aware moves (N=53 participants). **A, B.** Pre-movement positivity (PMP). Topoplots (**A**) show voltage at representative time points for significant clusters by spatiotemporal permutation statistics (see Supplemental Methods, $p<0.05$) for short-delay (2-5s) aware, long-delay (6-8s) aware, and short minus long delay conditions, with significant electrodes indicated by black outlines. Voltage timecourse data (**B**) for short-delay aware and long-delay aware trials averaged across participants (±SEM) from representative electrode within the significant PMP cluster (E5; right of Fz). Red line would indicate significant ($p<0.05$) short minus long delay time points in the spatiotemporal cluster analysis (no significant differences were seen). Time of move confirmation is t=0. **C, D.** Post-movement somatosensory perceptual N140 event-related potential. Topoplots (**C**) and voltage timecourse data (E155; posterior to C4) (**D**) displayed with same conventions as (**A, B**).

Supplementary Figure S7

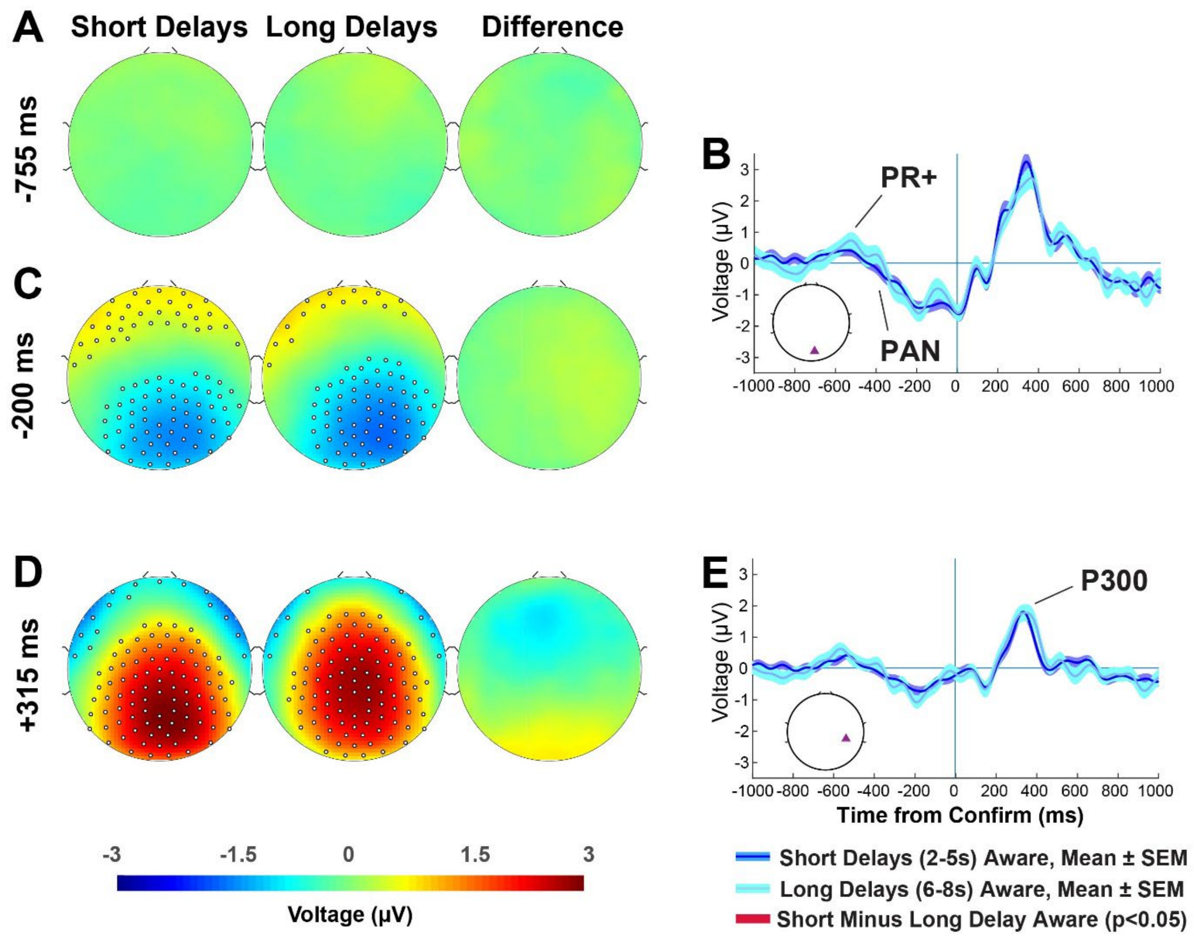

Supplementary Figure S7 (Related to Figure 3). Comparison of precursors and consequences for aware actions, short delays vs. long delays. Early and late event-related potentials relative to move confirmation on the Rush Hour game for short-delay aware and long-delay aware moves (N=53 participants). **A, B, C.** Pre-readiness positivity (PR+) and pre-action negativity. Topoplots show voltage at representative time points for the PR+ (**A**) and pre-action negativity (PAN) (**C**), with significant clusters by spatiotemporal permutation statistics (see Supplemental Methods, $p<0.05$) for short-delay aware, long-delay aware, and short minus long delay conditions. Significant electrodes are indicated by black outlines. Voltage timecourse data (**B**) for short-delay aware and long-delay aware trials averaged across participants (±SEM) from representative electrode within the significant PR+ cluster (E127, posterior to Pz), also showing timecourse of the pre-action negativity. Red line would indicate significant ($p<0.05$) short-delay minus long-delay time points in the spatiotemporal cluster analysis (no significant differences were seen).. Time of move confirmation is t=0. **D, E.** Post-perceptual P300 event-related potential (E164, posterior to C4). Topoplots (**D**) and voltage timecourse data (**E**) displayed with same conventions as (**A, B, C**).

Supplementary Figure S8

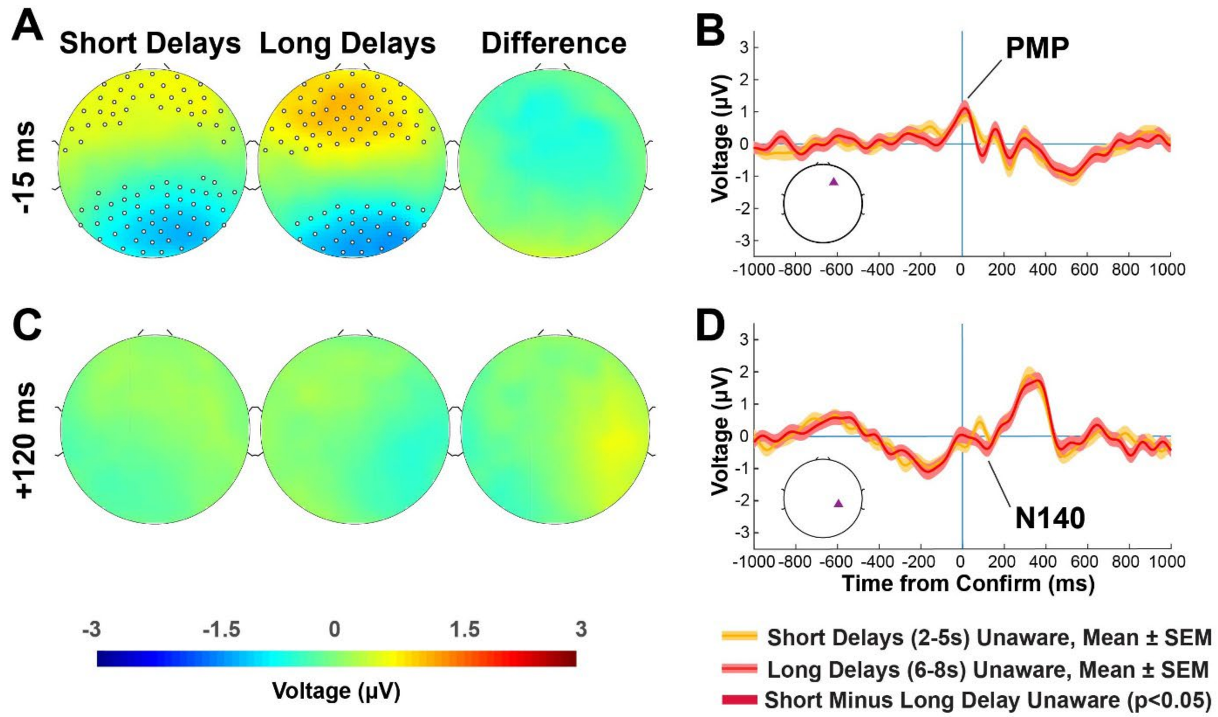

Supplementary Figure S8 (Related to Figure 2). Comparison of event-related potentials for unaware actions, short delays vs. long delays. Event-related potentials relative to move confirmation on the Rush Hour game for short-delay unaware and long-delay unaware moves (N=53 participants). **A, B.** Pre-movement positivity (PMP). Topoplots (**A**) show voltage at representative time points for significant clusters by spatiotemporal permutation statistics (see Supplemental Methods, p<0.05) for short-delay (2-5s) unaware, long-delay (6-8s) unaware, and short minus long delay conditions, with significant electrodes indicated by black outlines. Voltage timecourse data (**B**) for short-delay unaware and long-delay unaware trials averaged across participants (±SEM) from representative electrode within the significant PMP cluster (E5; right of Fz). Red line would indicate significant (p<0.05) short minus long delay time points in the spatiotemporal cluster analysis (no significant differences were seen). Time of move confirmation is t=0. **C, D.** Post-movement somatosensory perceptual N140 event-related potential. Topoplots (**C**) and voltage timecourse data (E155; posterior to C4) (**D**) displayed with same conventions as (**A, B**).

Supplementary Figure S9

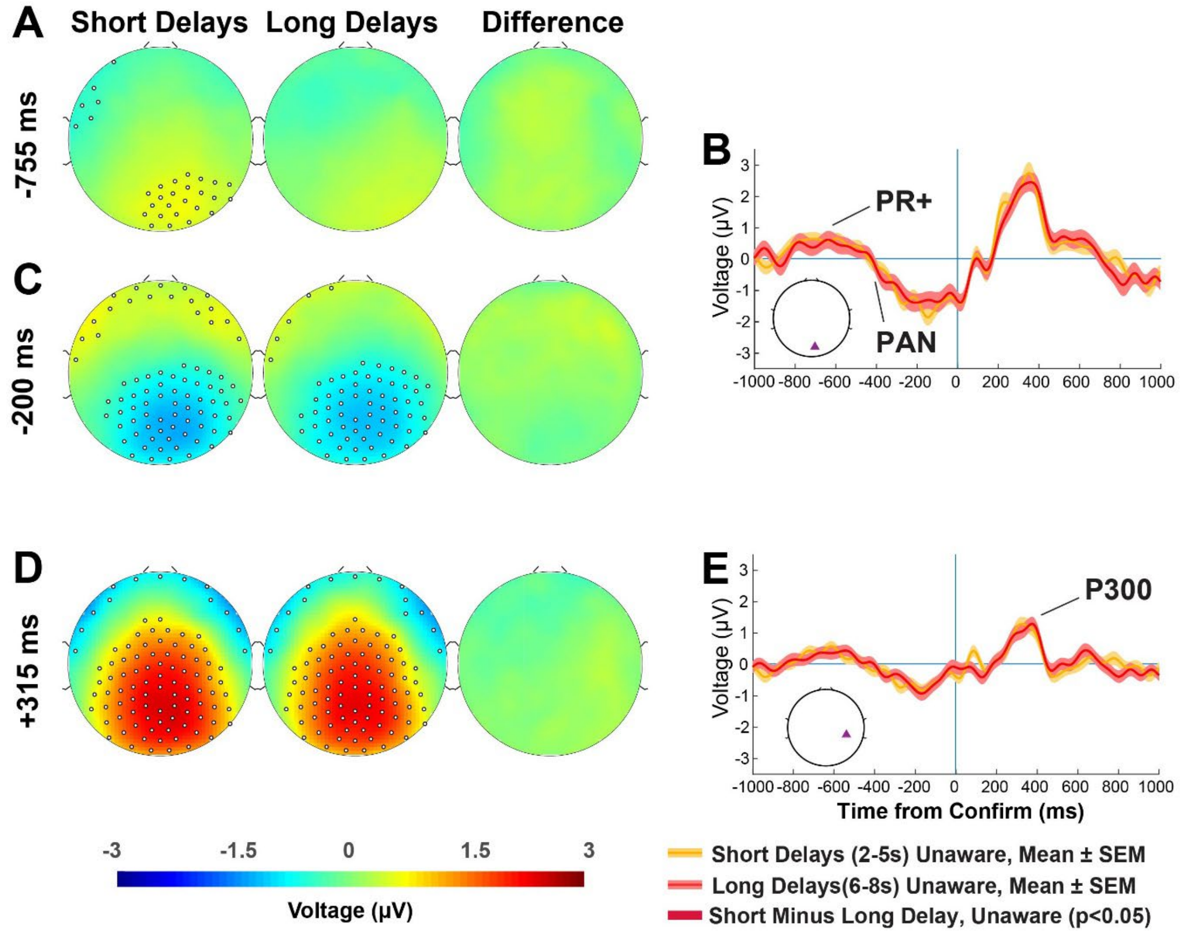

Supplementary Figure S9 (Related to Figure 3). Comparison of precursors and consequences for unaware actions, short delays vs. long delays (Related to Figure 3). Early and late event-related potentials relative to move confirmation on the Rush Hour game for short-delay unaware and long-delay unaware moves (N=53 participants). **A, B, C.** Pre-readiness positivity (PR+) and pre-action negativity (PAN). Topoplots show voltage at representative time points for the PR+ (**A**) and pre-action negativity (**C**), with significant clusters by spatiotemporal permutation statistics (see Supplemental Methods, $p<0.05$) for short-delay unaware, short-delay unaware, and short minus long delay conditions. Significant electrodes are indicated by black outlines. Voltage timecourse data (**B**) for short-delay unaware and long-delay unaware trials averaged across participants (±SEM) from representative electrode within the significant PR+ cluster (E127, posterior to Pz), also showing timecourse of the pre-action negativity. Red line would indicate significant ($p<0.05$) short-delay minus long-delay time points in the spatiotemporal cluster analysis (no significant differences were seen). Time of move confirmation is t=0. **D, E.** Post-perceptual P300 event-related potential (E164, posterior to C4). Topoplots (**D**) and voltage timecourse data (**E**) displayed with same conventions as (**A, B, C**).

Supplementary Figure S10

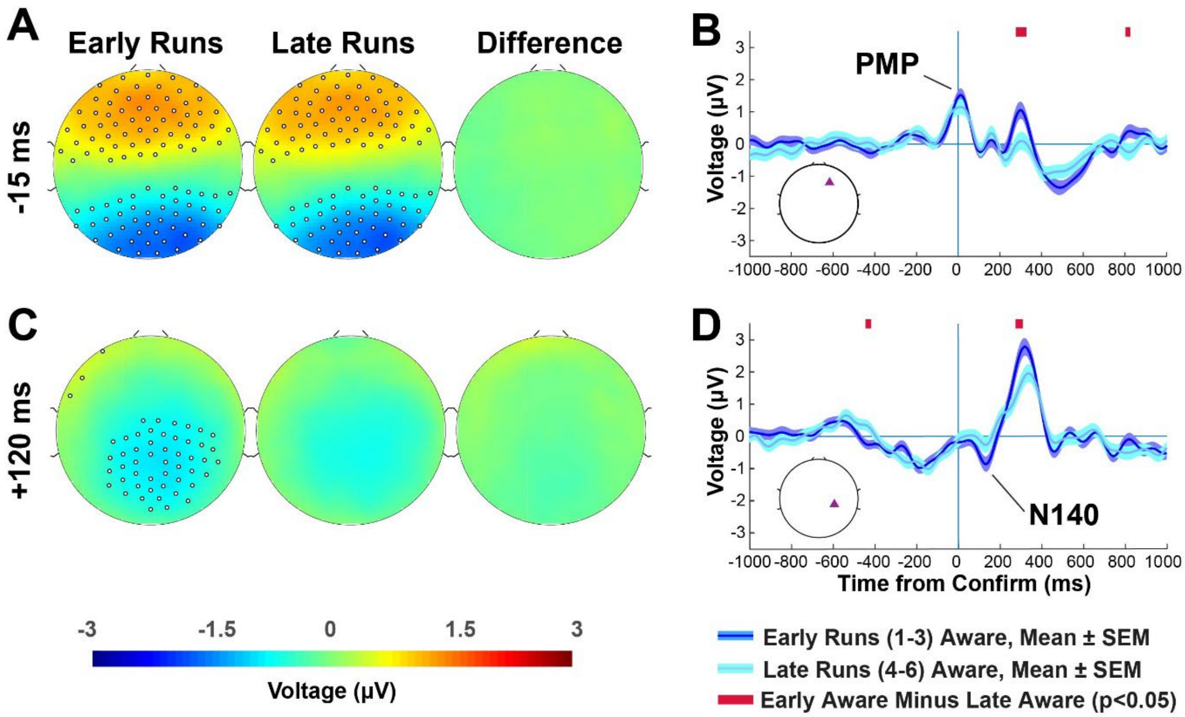

Supplementary Figure S10 (Related to Figure 2): Comparison of event-related potentials for aware actions, early runs vs. late runs. Event-related potentials relative to move confirmation on the Rush Hour game for early-run aware and late-run aware moves (N=57 participants). **A, B.** Pre-movement positivity (PMP). Topoplots (**A**) show voltage at representative time points for significant clusters by spatiotemporal permutation statistics (see Supplemental Methods, p<0.05) for early-run (runs 1-3) aware, late-run (runs 4-6) aware, and early minus late run conditions, with significant electrodes indicated by black outlines. Voltage timecourse data (**B**) for early-run aware and late-run aware trials averaged across participants (±SEM) from representative electrode within the significant PMP cluster (E5; right of Fz). Red line indicates significant (p<0.05) early minus late run time points in the spatiotemporal cluster analysis. Time of move confirmation is t=0. **C, D.** Post-movement somatosensory perceptual N140 event-related potential. Topoplots (**C**) and voltage timecourse data (E155; posterior to C4) (**D**) displayed with same conventions as (**A, B**).

Supplementary Figure S11

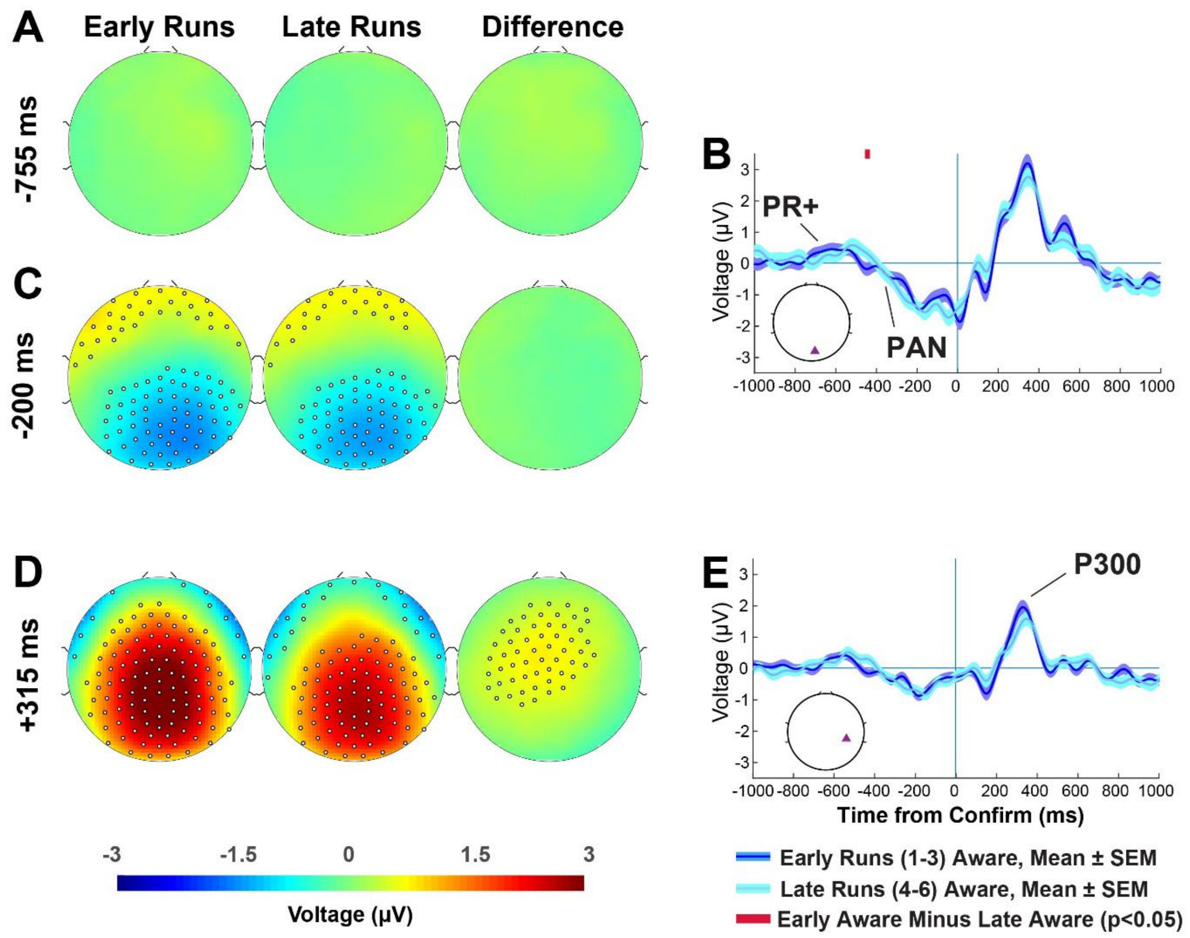

Supplementary Figure S11 (Related to Figure 3): Comparison of precursors and consequences for aware actions, early runs vs. late runs. Early and late event-related potentials relative to move confirmation on the Rush Hour game for early-run aware and late-run aware moves (N=57 participants). **A, B, C.** Pre-readiness positivity (PR+) and pre-action negativity. Topoplots show voltage at representative time points for the PR+ (**A**) and pre-action negativity (PAN) (**C**), with significant clusters by spatiotemporal permutation statistics (see Supplemental Methods, $p<0.05$) for early-run aware, late-run aware, and early minus late run conditions. Significant electrodes are indicated by black outlines. Voltage timecourse data (**B**) for early-run aware and late-run aware trials averaged across participants (±SEM) from representative electrode within the significant PR+ cluster (E127, posterior to Pz), also showing timecourse of the pre-action negativity. Red line indicates significant ($p<0.05$) early-run minus late-run time points in the spatiotemporal cluster analysis. Time of move confirmation is t=0. **D, E.** Post-perceptual P300 event-related potential (E164, posterior to C4). Topoplots (**D**) and voltage timecourse data (**E**) displayed with same conventions as (**A, B, C**).

Supplementary Figure S12

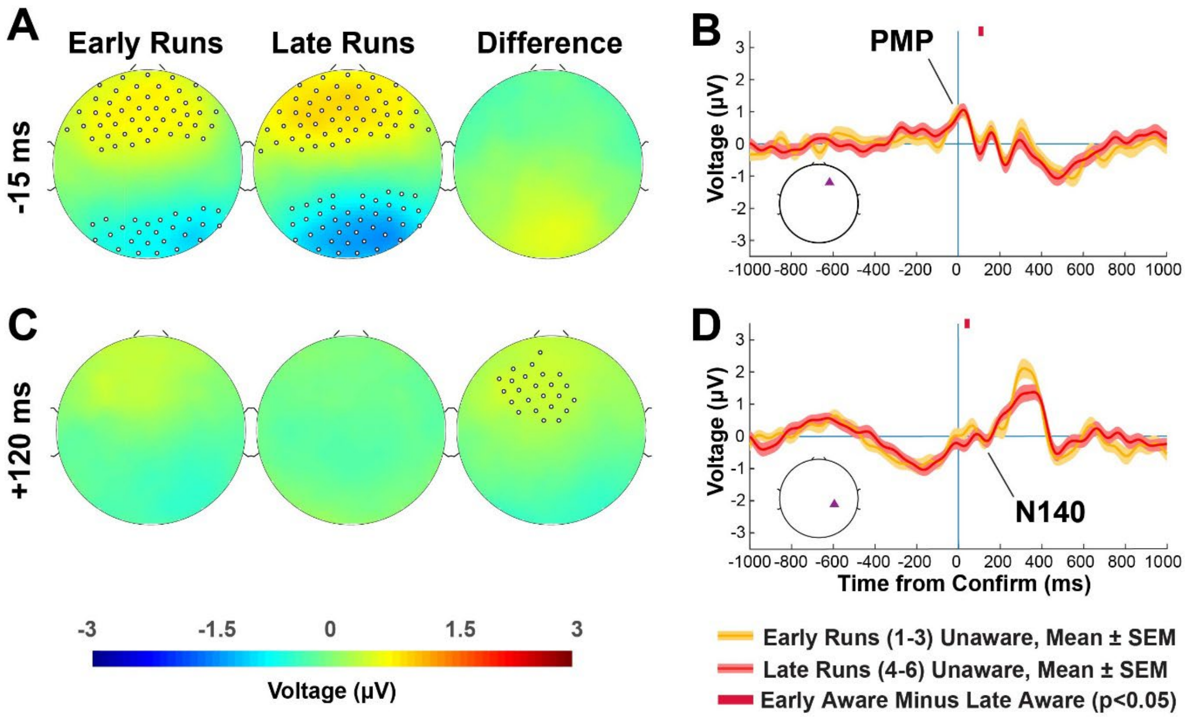

Supplementary Figure S12 (Related to Figure 2). Comparison of event-related potentials for unaware actions, early runs vs. late runs. Event-related potentials relative to move confirmation on the Rush Hour game for early-run unaware and early-run unaware moves (N=57 participants). **A, B.** Pre-movement positivity (PMP). Topoplots (**A**) show voltage at representative time points for significant clusters by spatiotemporal permutation statistics (see Supplemental Methods, $p<0.05$) for early-run (runs 1-3) unaware, late-run (runs 4-6) unaware, and early minus late run conditions, with significant electrodes indicated by black outlines. Voltage timecourse data (**B**) for early-run unaware and late-run unaware trials averaged across participants (±SEM) from representative electrode within the significant PMP cluster (E5; right of Fz). Red line indicates significant ($p<0.05$) early minus late run time points in the spatiotemporal cluster analysis. Time of move confirmation is t=0. **C, D.** Post-movement somatosensory perceptual N140 event-related potential. Topoplots (**C**) and voltage timecourse data (E155; posterior to C4) (**D**) displayed with same conventions as (**A, B**).

Supplementary Figure S13

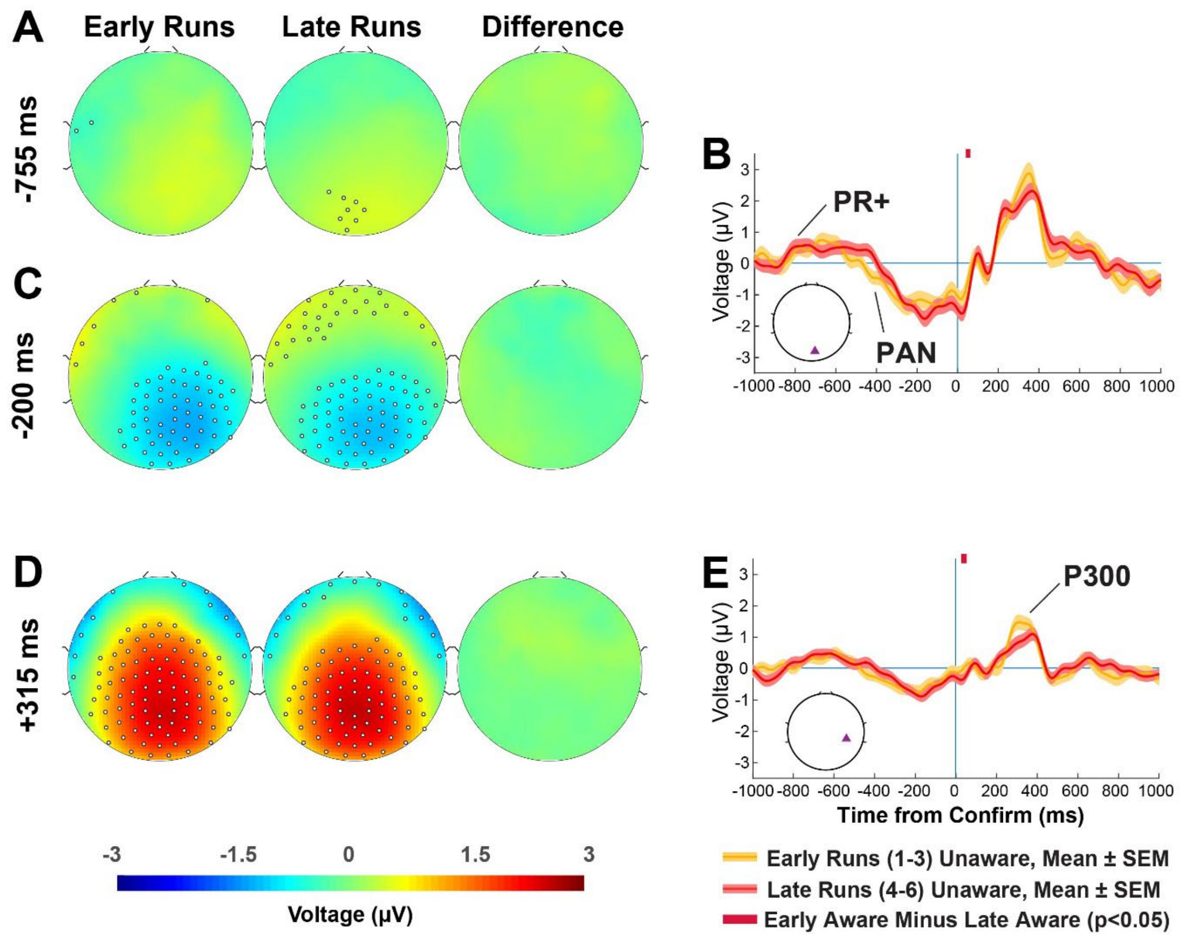

Supplementary Figure S13 (Related to Figure 3). Comparison of precursors and consequences for unaware actions, early runs vs. late runs. Early and late event-related potentials relative to move confirmation on the Rush Hour game for early-run unaware and late-run unaware moves (N=57 participants). **A, B, C.** Pre-readiness positivity (PR+) and pre-action negativity. Topoplots show voltage at representative time points for the PR+ (**A**) and pre-action negativity (PAN) (**C**), with significant clusters by spatiotemporal permutation statistics (see Supplemental Methods, $p<0.05$) for early-run unaware, late-run unaware, and early minus late run conditions. Significant electrodes are indicated by black outlines. Voltage timecourse data (**B**) for early-run unaware and late-run unaware trials averaged across participants (±SEM) from representative electrode within the significant PR+ cluster (E127, posterior to Pz), also showing timecourse of the pre-action negativity. Red line indicates significant ($p<0.05$) early-run minus late-run time points in the spatiotemporal cluster analysis. Time of move confirmation is t=0. **D, E.** Post-perceptual P300 event-related potential (E164, posterior to C4). Topoplots (**D**) and voltage timecourse data (**E**) displayed with same conventions as (**A, B, C**).

Supplementary Figure S14

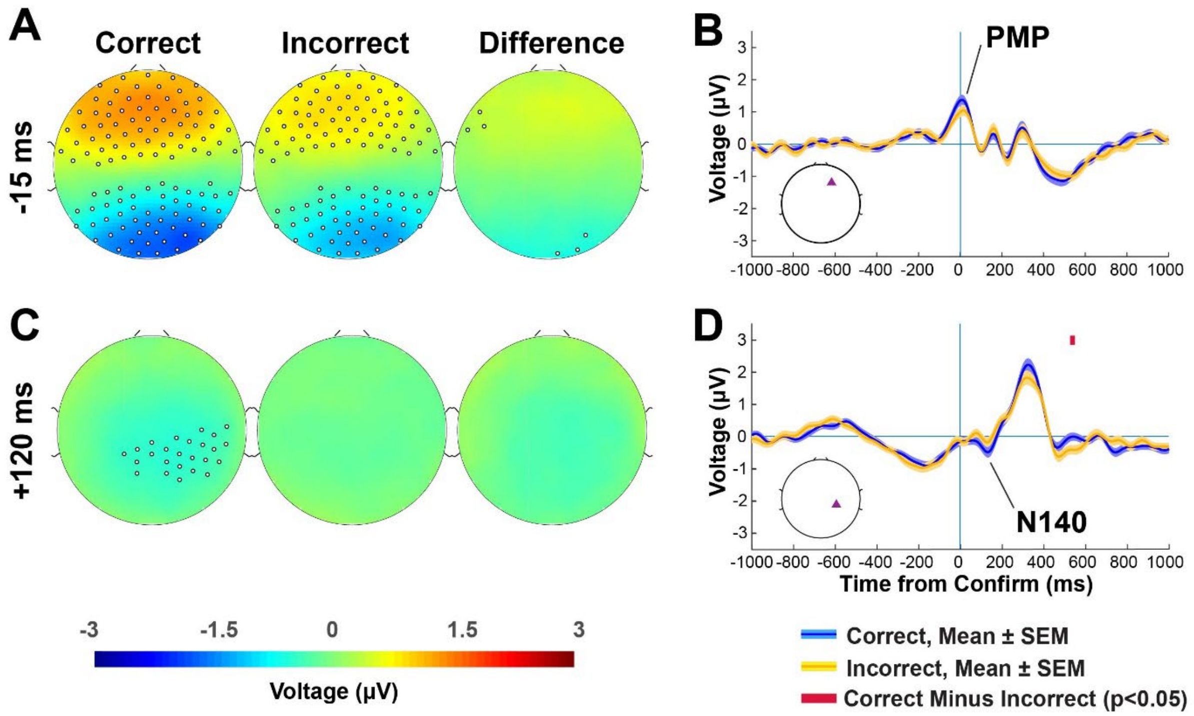

Supplementary Figure S14. Comparison of event-related potentials for correctly and incorrectly identified actions (Related to Figure 2). Event-related potentials relative to move confirmation on the Rush Hour game for correct and incorrect moves (N=57 participants). **A, B.** Pre-movement positivity (PMP). Topoplots (**A**) show voltage at representative time points for significant clusters by spatiotemporal permutation statistics (see Supplemental Methods, $p<0.05$) for correct, incorrect, and correct minus incorrect conditions, with significant electrodes indicated by black outlines. Voltage timecourse data (**B**) for correct and incorrect trials averaged across participants (±SEM) from representative electrode within the significant PMP cluster (E5; right of Fz). Red line indicates significant ($p<0.05$) correct minus incorrect time points in the spatiotemporal cluster analysis. Time of move confirmation is t=0. **C, D.** Post-movement somatosensory perceptual N140 event-related potential. Topoplots (**C**) and voltage timecourse data (E155; posterior to C4) (**D**) displayed with same conventions as (**A, B**).

Supplementary Figure S15

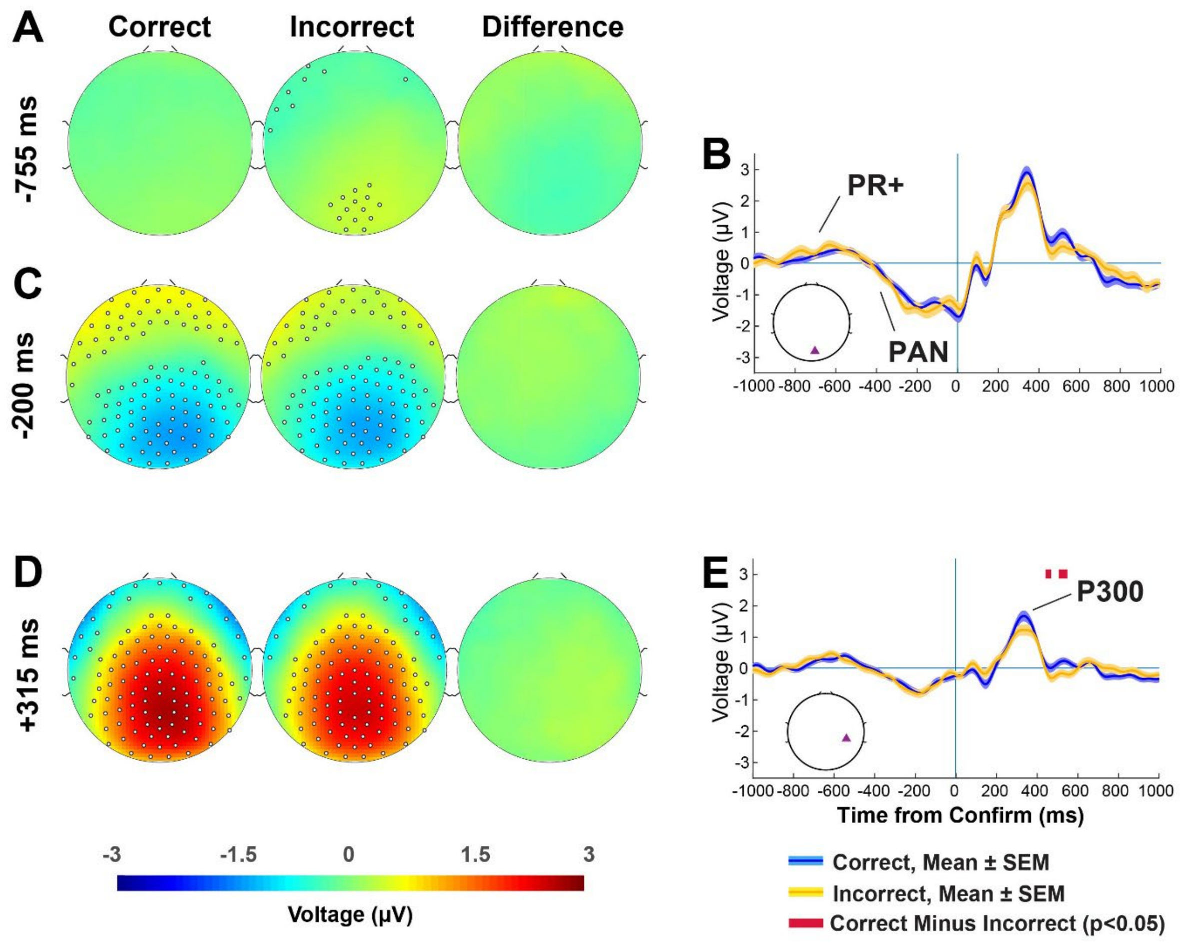

Supplementary Figure S15 (Related to Figure 3). Comparison of precursors and consequences for correctly and incorrectly identified actions. Early and late event-related potentials relative to move confirmation on the Rush Hour game for correct and incorrect moves (N=57 participants). **A, B, C.** Pre-readiness positivity (PR+) and pre-action negativity. Topoplots show voltage at representative time points for the PR+ (**A**) and pre-action negativity (PAN) (**C**), with significant clusters by spatiotemporal permutation statistics (see Supplemental Methods, $p<0.05$) for correct, incorrect, and correct minus incorrect conditions. Significant electrodes are indicated by black outlines. Voltage timecourse data (**B**) for correct and incorrect trials averaged across participants (±SEM) from representative electrode within the significant PR+ cluster (E127, posterior to Pz), also showing timecourse of the pre-action negativity. Red line indicates significant ($p<0.05$) correct minus incorrect time points in the spatiotemporal cluster analysis. Time of move confirmation is t=0. **D, E.** Post-perceptual P300 event-related potential (E164, posterior to C4). Topoplots (**D**) and voltage timecourse data (**E**) displayed with same conventions as (**A, B, C**).

Supplementary Figure S16

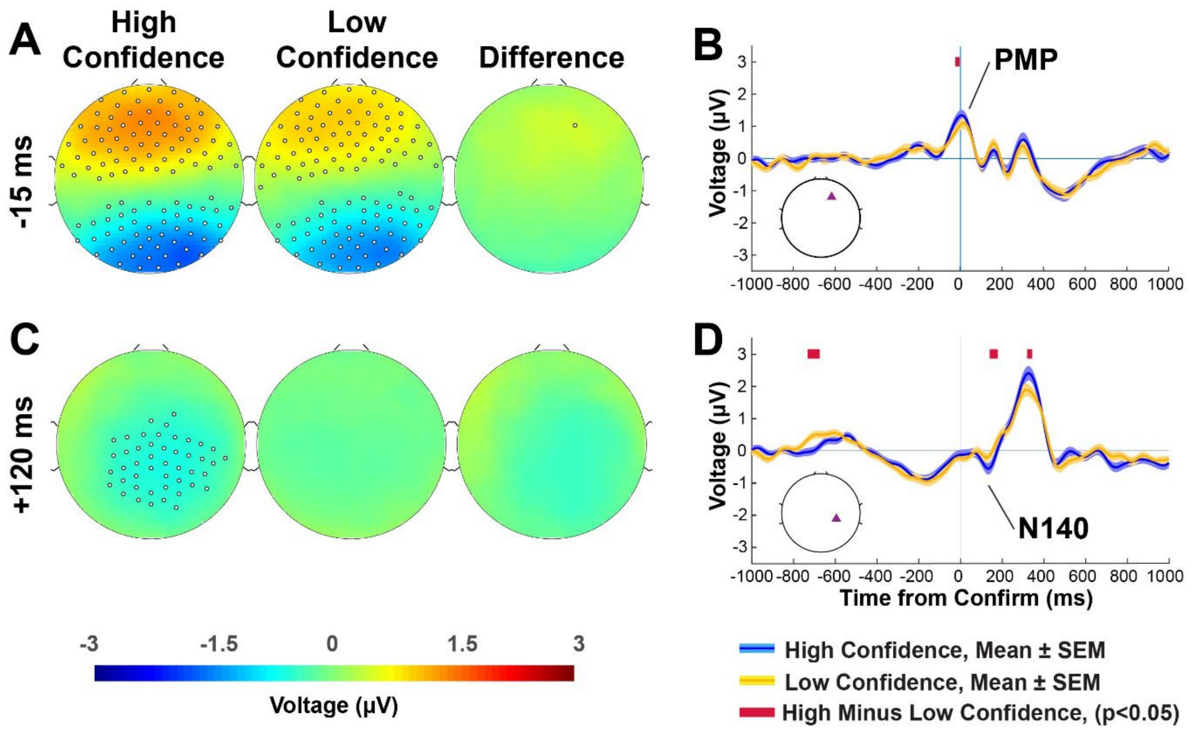

Supplementary Figure S16 (Related to Figure 2). Comparison of event-related potentials for high-confidence and low-confidence actions. Event-related potentials relative to move confirmation on the Rush Hour game for high-confidence (>75th percentile) and low-confidence (<25th percentile) moves (N=57 participants). **A, B.** Pre-movement positivity (PMP). Topoplots (**A**) show voltage at representative time points for significant clusters by spatiotemporal permutation statistics (see Supplemental Methods, p<0.05) for high confidence, low confidence, high minus low confidence conditions, with significant electrodes indicated by black outlines. Voltage timecourse data (**B**) for high confidence and low confidence trials averaged across participants (±SEM) from representative electrode within the significant PMP cluster (E5; right of Fz). Red line indicates significant (p<0.05) high confidence minus low confidence time points in the spatiotemporal cluster analysis. Time of move confirmation is t=0. **C, D.** Post-movement somatosensory perceptual N140 event-related potential. Topoplots (**C**) and voltage timecourse data (E155; posterior to C4) (**D**) displayed with same conventions as (**A, B**).

Supplementary Figure S17

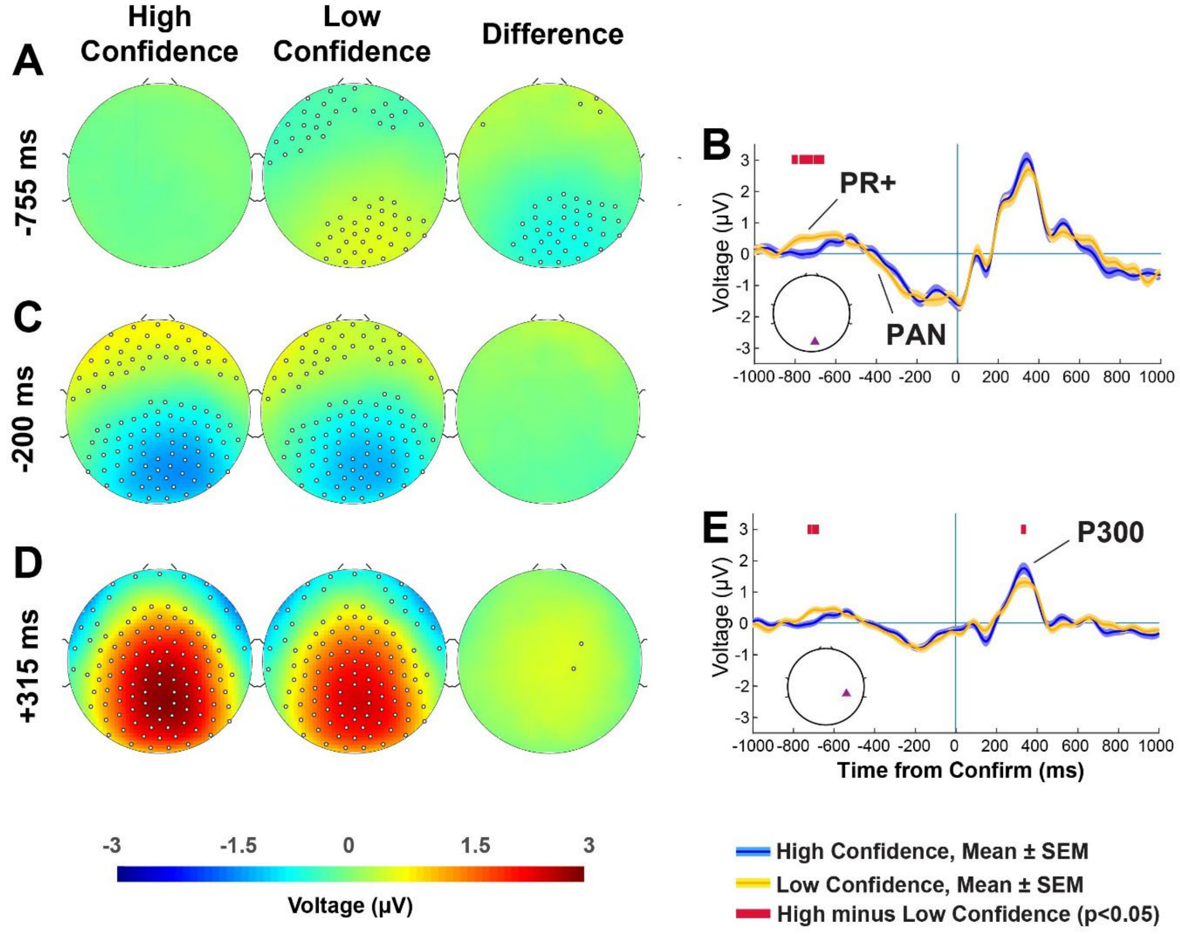

Supplementary Figure S17 (Related to Figure 3). Comparison of precursors and consequences for high-confidence and low-confidence actions. Early and late event-related potentials relative to move confirmation on the Rush Hour game for high confidence (>75th percentile) and low confidence (<25th percentile) moves (N=57 participants). **A, B, C.** Pre-readiness positivity (PR+) and pre-action negativity (PAN). Topoplots show voltage at representative time points for the PR+ (**A**) and pre-action negativity (**C**), with significant clusters by spatiotemporal permutation statistics (see Supplemental Methods, p<0.05) for high confidence, low confidence, and high minus low confidence conditions. Significant electrodes are indicated by black outlines. Voltage timecourse data (**B**) for high confidence and low confidence trials averaged across participants (±SEM) from representative electrode within the significant PR+ cluster (E127, posterior to Pz), also showing timecourse of the pre-action negativity. Red line indicates significant (p<0.05) high confidence minus low confidence time points in the spatiotemporal cluster analysis. Time of move confirmation is t=0. **D, E.** Post-perceptual P300 event-related potential (E164, posterior to C4). Topoplots (**D**) and voltage timecourse data (**E**) displayed with same conventions as (**A, B, C**).

Supplementary Figure S18

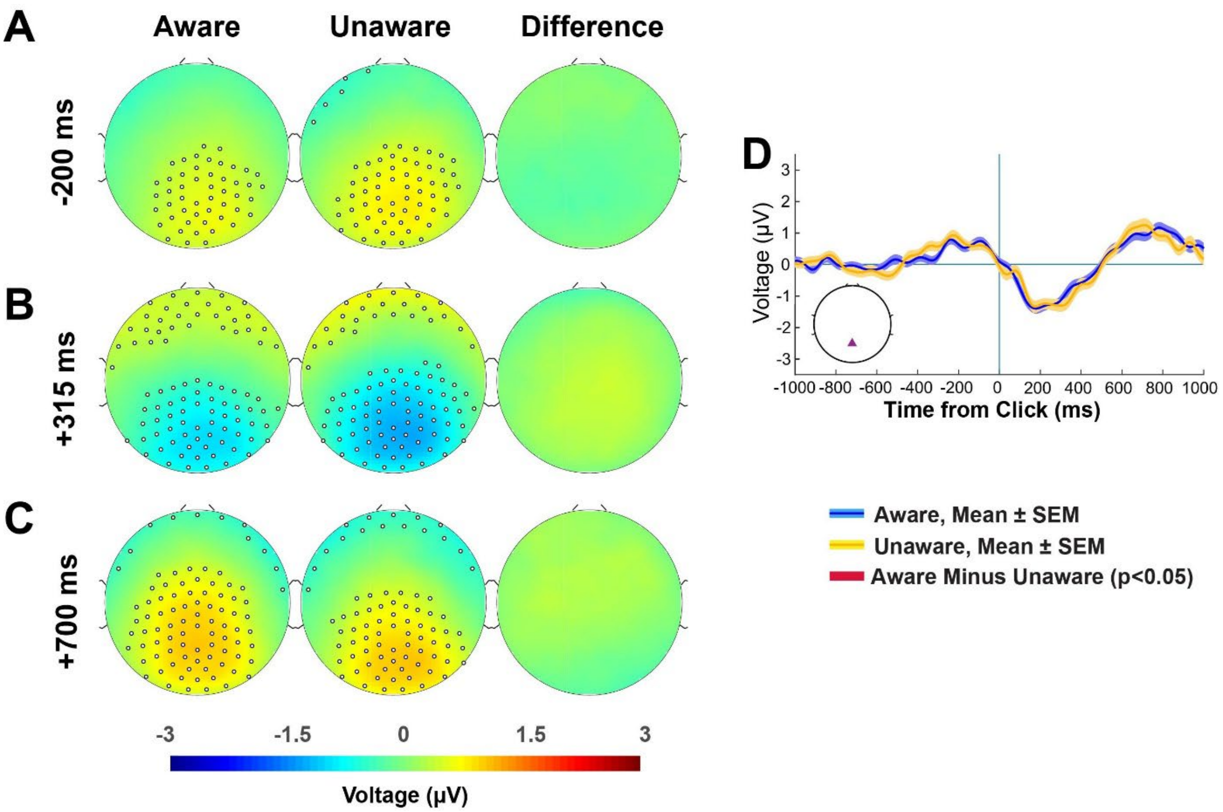

Supplementary Figure S18. Comparison of aware and unaware actions time-locked to the selection of a block. **A**. Prior to the action, a slow-moving positivity is observed, followed by **B.** a negative deflection, coincident with the pre-confirm negativity (see Figure 3C). Finally, a **C.** positivity occurring ~700 ms post-action, temporally coincident with the P300 associated with the confirmation of the action (see Figure 3D). **D.** A representative timecourse (E101; Pz). Significant electrodes in (**A**), (**B**), and (**C**) are indicated by black outlines. Voltage timecourse data (**D**) for aware and unaware trials averaged across participants (±SEM). No significant differences (red bar) are seen between aware and unaware trials.

Supplementary Figure S19

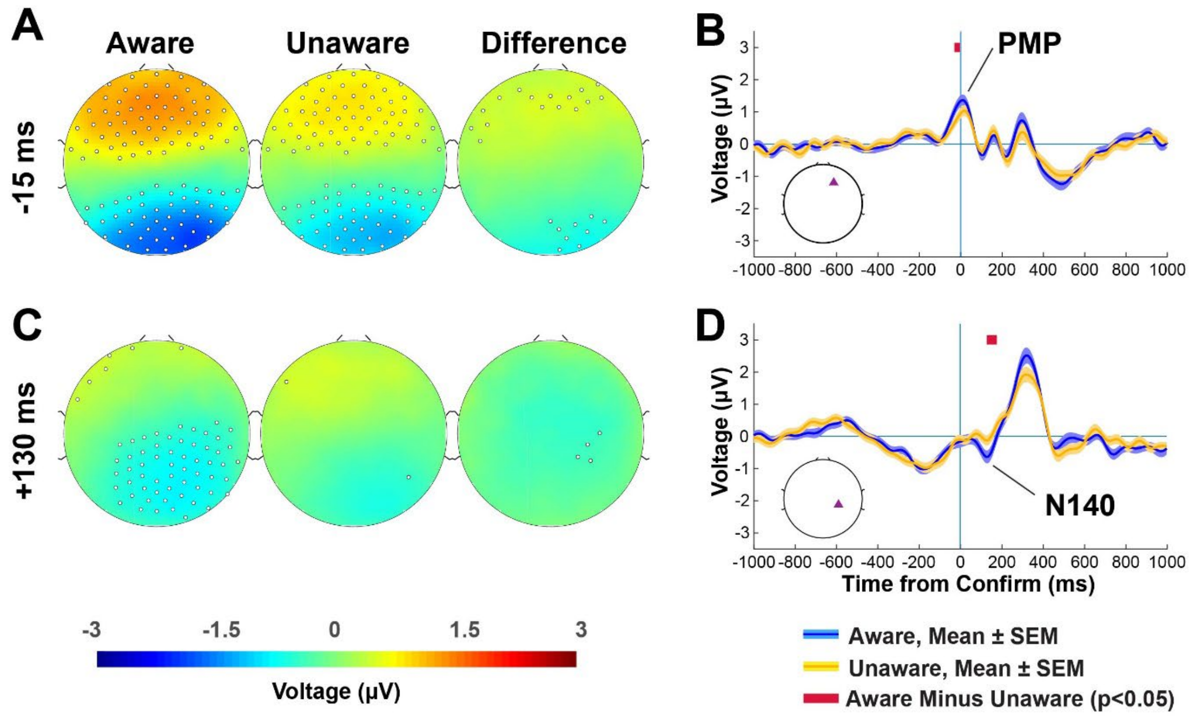

Supplementary Figure S19 (Related to Figure 2). Comparison of event-related potentials for aware and unaware actions following trial counterbalancing. Event-related potentials relative to move confirmation on the Rush Hour game for aware and unaware moves when trial counts were balanced within each participant via bootstrapping (N=57 participants). **A, B.** Pre-movement positivity (PMP). Topoplots (**A**) show voltage at representative time points for significant clusters by spatiotemporal permutation statistics (see Supplemental Methods, p<0.05) for aware, unaware, aware minus unaware conditions, with significant electrodes indicated by black outlines. Voltage timecourse data (**B**) for aware and unaware trials averaged across participants (±SEM) from representative electrode within the significant PMP cluster (E5; right of Fz). Red line indicates significant (p<0.05) aware minus unaware time points in the spatiotemporal cluster analysis. Time of move confirmation is t=0. **C, D.** Post-movement somatosensory perceptual N140 event-related potential. Topoplots (**C**) and voltage timecourse data (E155; posterior to C4) (**D**) displayed with same conventions as (**A, B**).

Supplementary Figure S20

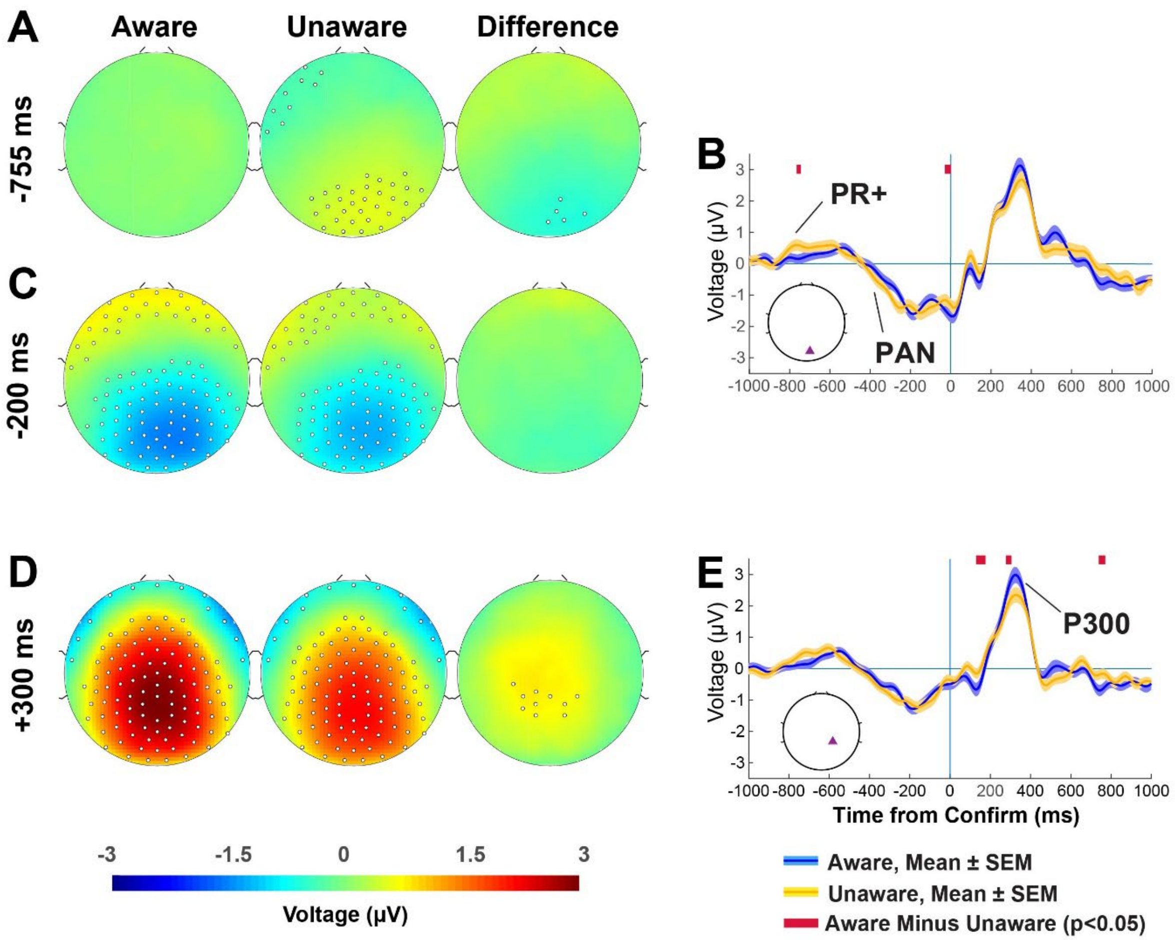

Supplementary Figure S20 (Related to Figure 3). Comparison of precursors and consequences for aware and unaware actions following trial counterbalancing. Early and late event-related potentials relative to move confirmation on the Rush Hour game for aware and unaware moves when trial counts were balanced within each participant via bootstrapping (N=57 participants). **A, B, C.** Pre-readiness positivity (PR+) and pre-action negativity (PAN). Topoplots show voltage at representative time points for the PR+ (**A**) and pre-action negativity (**C**), with significant clusters by spatiotemporal permutation statistics (see Supplemental Methods, $p<0.05$) for aware, unaware, and aware minus unaware conditions. Significant electrodes are indicated by black outlines. Voltage timecourse data (**B**) for aware and unaware trials averaged across participants (±SEM) from representative electrode within the significant PR+ cluster (E127, posterior to Pz), also showing timecourse of the pre-action negativity. Red line indicates significant ($p<0.05$) aware minus unaware time points in the spatiotemporal cluster analysis. Time of move confirmation is t=0. **D, E.** Post-perceptual P300 event-related potential (E143, posterior to C4). Topoplots (**D**) and voltage timecourse data (**E**) displayed with same conventions as (**A, B, C**).

Supplementary Figure S21

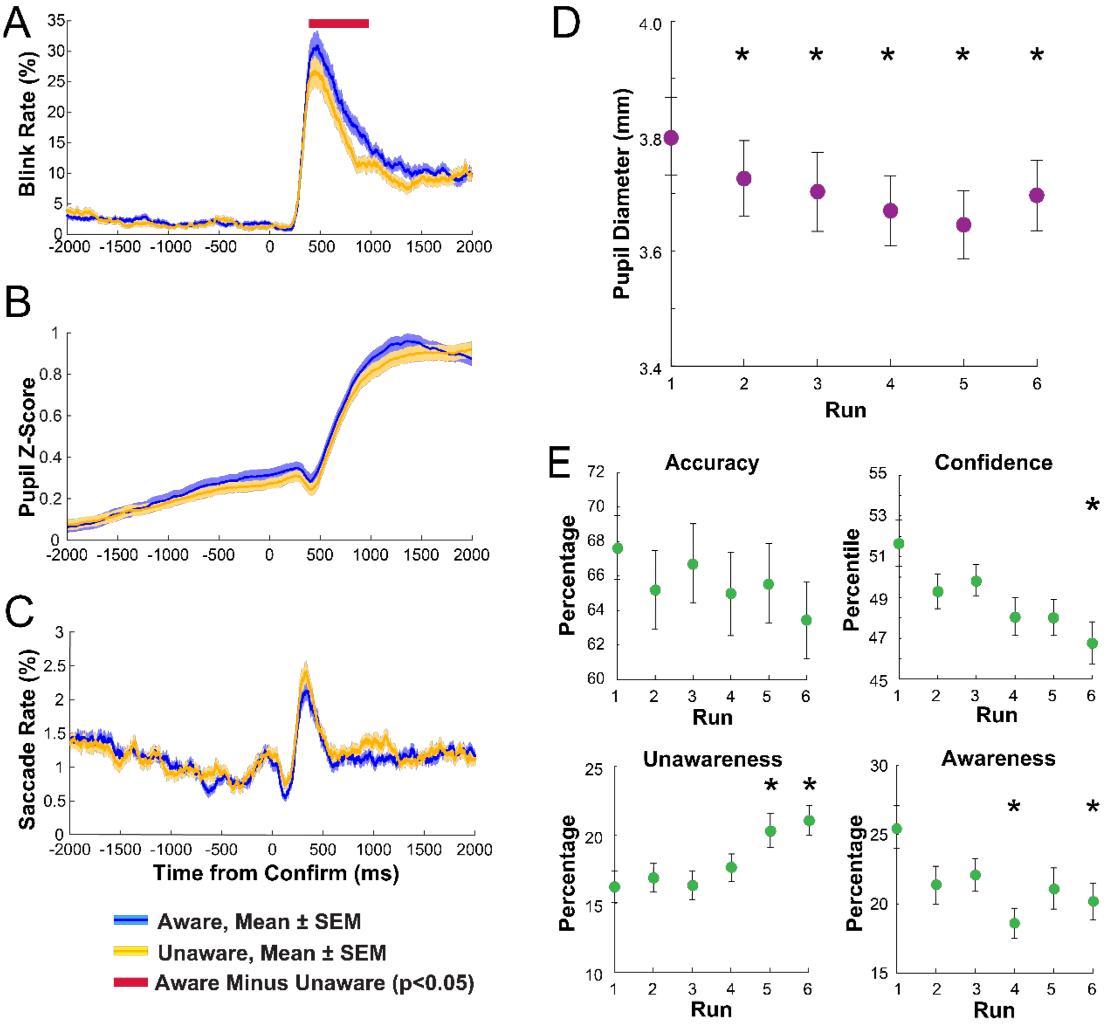

Supplementary Figure S21 Eye metrics and long-term behavioral metrics in relation to AoA. A, B, C. Perception-related eye metrics (N = 56 participants). **A.** Blink rates. **B.** Pupil diameter. **C.** Saccade rates. Aware and unaware trials were averaged within participant, then across participants, and permutation statistics performed on the group level. Red line indicates significant aware minus unaware time points ($p<0.05$, see Supplemental Methods). Time of move confirmation is t=0. **D.** Run-by-run averages of raw pupil diameter ± SEM (N=56 participants). **E.** Run-by-run averages of unawareness rate, awareness rate, quiz accuracy, and confidence percentile ± SEM (N=67 participants). **D, E.** Significance of each run was assessed with a paired two-tailed t-test against Run 1 (* $p<0.05$, corrected with the Benjamini-Hochberg FDR procedure).

Supplementary Figure S22

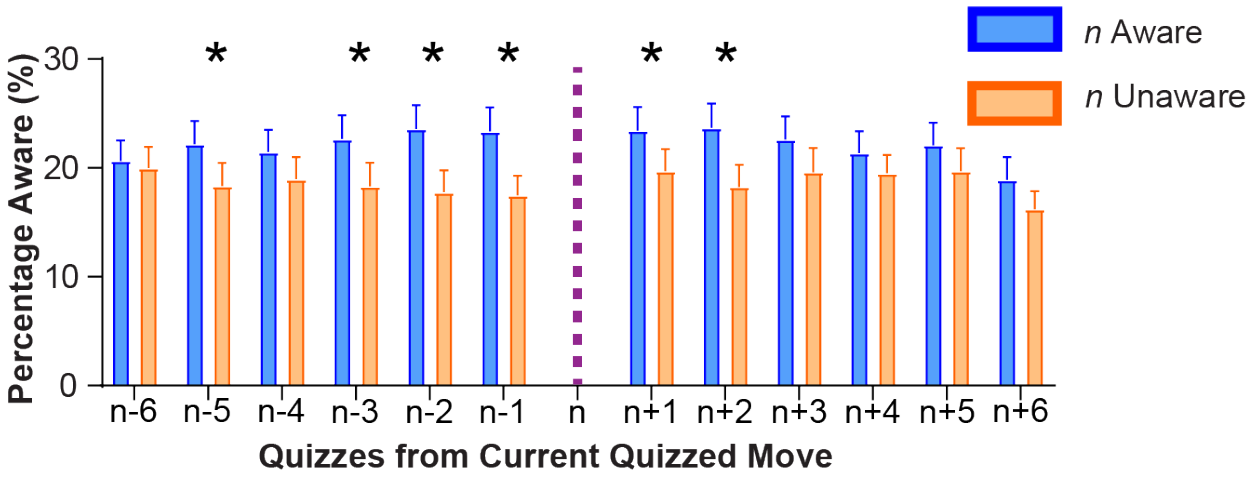

Supplementary Figure S22 Awareness rates surrounding a move that is aware versus a move that is unaware. Awareness rates (% of moves) were calculated by averaging within participants and then across participants (N=67 participants). Mean ± SEM awareness rates are shown for quizzed moves preceding and following a designated quizzed move *n* (vertical dashed line), where the move n was either aware (blue) or unaware (orange). Statistical significance was assessed for each of the 12 surrounding quizzed moves n-6 to n+6 versus move n with a two-tailed paired t-test (*p<0.05 with Benjamini-Hochberg FDR correction).

Supplementary Figure S23

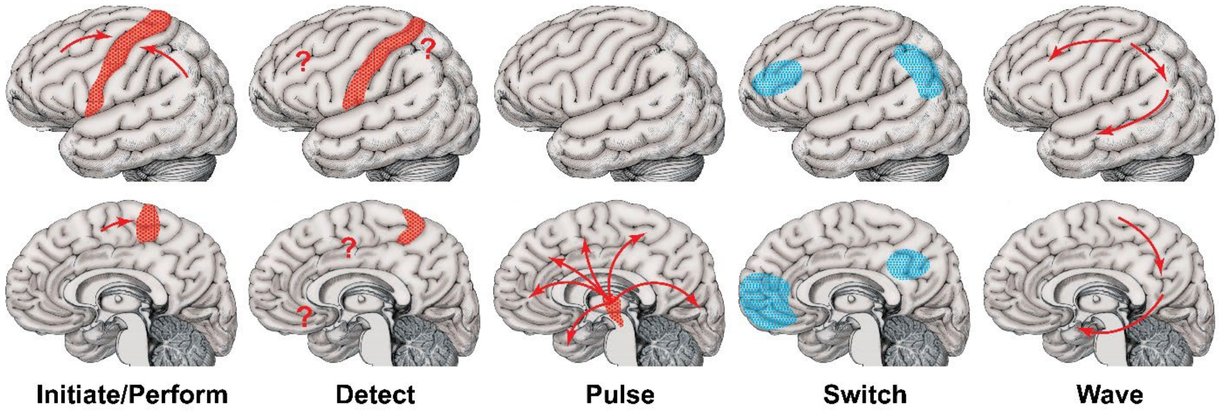

Supplementary Figure S23 Combined volitional and perceptual model of AoA. Preparation of actions is generated and initiated in supplementary and presupplementary motor cortices and is performed together with primary motor cortex (Initiate/Perform). Next, initial somatosensory detection of the action occurs in primary somatosensory cortex and connected higher-order detection areas (Detect). Following the detection, a transient pulse of arousal from subcortical regions amplifies signals broadly, facilitating subsequent perceptual processing for AoA. To focus resources on relevant processing, activity in the default mode network and other regions is switched off to devote neural resources to the processing of the action (Switch). Lastly, a wave of activity in widespread association cortex regions allows for the full processing of AoA as well as encoding in memory circuits and subsequent report/description of AoA (Wave). Modified with permission from [5]. See also **Supplementary Table S6**.

Supplementary Video S1 Recorded example of game play with simultaneous background video. Sequence begins with two moves that lead to successful completion of the block slider puzzle, while video plays in the background. Next there are three more moves, followed by disappearance of the game board, then a pause, and finally responses to the two part quiz including correct identification of the last move and a relatively high confidence rating.

Supplemental Methods

Participants

Seventy-seven clinically healthy, adult participants were recruited and tested for our study. Inclusion criteria were normal vision (with soft lens correction) and normal hearing. Exclusion criteria were current or past diagnoses of neurological disorders and vision correction which required hard lenses (because this would interfere with eye measurements). After data acquisition ten participants were excluded based on data quality criteria (next section), leaving a total of 67 participants for analysis (43 female, 24 male), with an average age of 22 ± 0.38 (mean ± SEM) years.

Data Quality Exclusion Criteria

Behavioral exclusion criteria were applied to ensure that the data analyzed for each participant included a minimum of 12 aware and 12 unaware trials (see next section for definitions). The 12 trial minimum was applied separately to EEG and eye metric data for each participant, and was applied after application of any other data quality exclusion criteria (see *EEG – Data Preprocessing,* and *Eye Metrics – Individual Trial Rejection*). With these criteria, of the initial 77 participants enrolled and tested, 10 were excluded due to insufficient number of trials for both EEG and eye metric data, leaving a total of 67 participants for analysis. Of these, the final sample sizes with sufficient trials for analysis were 67 participants for behavioral data, 57 participants for EEG data, and 55 participants for eye metric data (45 for both EEG and eye metrics; 12 for EEG only; 10 for eye metrics only).

Behavioral Tasks

Equipment and Hardware

Participants completed the task using a gameplay mouse (Lenovo Essential USB Mouse) and keyboard (Lenovo Essential Wired Keyboard) attached via USB to the behavioral task laptop (**Supplementary Fig S1**). Participants viewed the game on a 17-inch LCD monitor on an arm mount (EyeLink 1000 Plus System, SR Research, Inc.) displaying the behavioral task laptop (MSI Gf63 Thin, MSI) visual feed located 55 cm. from the participant. The LCD screen was connected to the behavioral laptop via a VGA/HDMI adapter. The task code was implemented in the *PsychoPy* package of Python. Timing synchronization of the behavioral, EEG and eye metric data is described in a separate section below.

Task Design

The overall task design includes a slider puzzle game based on the well-known board game Rush Hour, and a distractor memory task consisting of background videos (**Fig 1A**). Participants were instructed to remember as much detail as possible from the videos while simultaneously playing the slider game. Periodically the game was interrupted with questions to assess whether or not participants were aware of their most recent game move (**Fig 1A**). The experimental design with a repetitive overlearned game in parallel with an engaging distractor task was intended to encourage participants to play the game at times without awareness.

Participants performed the task across three days, with one training day and two testing days (**Fig 1B**). Six ten-minute runs were administered on each day for a total of one hour of gametime per day. To complete the task, participants had to navigate a red block out of the bounds of a grid, by moving obstructing blocks to free a path for the block (**Fig 1A**; see also **Supplementary Video**

S1). To move a block, participants used the mouse in the right hand to click on and select a block. They then used the WASD keys in the left hand (W = up, middle finger; A = left, ring finger; S = down, middle finger; D = right, index finger) to move a block. Upon completion of the movement, participants pressed the spacebar with the left thumb to confirm the action. Participants could not select and move another block without first confirming the current block movement. Event related potential, time-frequency, and pupillometry analyses were time-locked to the confirmation of the action, and all further uses of "time from action" refer to timeframe with respect to the confirmation. When the participant solved an individual puzzle, a message depicting "Good Job" would appear on the right third of the screen, after which the next puzzle configuration would be shown. A randomized rotation of ten unique puzzle configurations was used. The ten puzzles were chosen after extensive pilot testing with more challenging and less challenging puzzles, to identify puzzle size and complexity that could be solved without difficulty, yet were complex enough to not be trivial, and therefore yielded a reasonable balance of aware and unaware moves in most participants. After every 2-5 moves (randomly varied), the board disappeared for a period of 2-8 seconds (random flat distribution with 1s intervals) before reappearing. Participants were initially trained on this task for three runs with a plain blue background (**Fig 1B**).

In latter three runs of the training day (Day 1), participants performed the task with a distractor free recall task added, in which they were instructed that at the end of each run, they would have to recall as many details as possible in a background video that played as they performed the task. The videos included audio and consisted of 15 (three practice on Day 1, 12 testing on Days 2-3) different short segments lasting up to 10 minutes, presented in random sequence with one

video per run. The videos were selected from YouTube, and were chosen to cover a wide breadth of subject matter to engage participants of varying academic and social backgrounds. The slider puzzle task occupied a rectangular area measuring 23.8 × 23.8 degrees of visual angle in the center of the screen, allowing the videos to be viewed around the sides of the screen. Participants were instructed to maintain a chosen pace of play even with the background video playing. At the end of each run, participants completed a three-minute recall session in which they were instructed to continue recounting details from the video until the end of the three minute period. Participants completed three runs of the game + free recall task on Day 1 (**Fig 1B**). The two testing days (Days 2 and 3) were identical to one another. On the testing days, the game with video background and free recall sessions were administered as previously. However, after the board disappeared following 2-5 moves, there was a 50% probability that a two-part quiz would be shown. The first part of the quiz was a four-part multiple choice question, in which four candidate moves (with the block and direction indicated by a black arrow) were presented to the participant with the instruction to "Choose your last move." (**Fig 1A**). These candidate moves were drawn from a large library of possible moves constructed from pilot testing of game play, and always included the participant's correct last move, placed in random location on the screen. Participants selected their last move by mouse click, forced choice with a three second time limit. The second part of a quiz was a confidence indication. Participants were asked to indicate their confidence level ("How certain are you of your choice?") by clicking on a scale bar (leftmost = least confident, rightmost = most confident), with no time limit on responses. Participants then pressed a "Go" key to submit their answer, and the game with video resumed after a delay of 5 to 7 seconds.

We found it was necessary to impose the three second time limit on responses to the multiple choice quiz to discourage participants from trying to logically recalculate a particular route taken in the game, as opposed to immediately demonstrating awareness of the previous action. If participants did not answer the multiple choice question within the three second limit, the confidence question was not administered and the trial was excluded from analysis. To improve compliance, during the first three runs, the total number of late trials were tabulated. If a participant averaged two or more late quizzes per run, or had three or more late quizzes in a single run, they were instructed, “Please provide an answer within the three second limit”.

Behavioral Data Acquisition

After each run, five types of behavioral data were saved, which enabled all behavioral metrics to be calculated: 1. Synchronization TTL pulse timing, 2. Multiple choice accuracy for each quiz, 3. Confidence slider positions for each quiz, 3. Order of puzzles presented to the subject, 4. Initial configurations prior to each move being performed, and 5. Background movie identity. These were saved in a *.psydat* file, constituting the behavioral data files from which awareness was determined.

Eye Tracking and Pupillometry

Equipment and Software

Pupillometry and eye metric data were collected using the EyeLink 1000 Plus System (v5.09, SR Research) running on a Dell desktop PC (Model D13M; Dell, Inc.). Data were sampled in binocular mode at 1000Hz using a 35 mm camera and an infrared illuminator mounted below the LCD game monitor (**Supplementary Fig S1**). Prior to the first and fourth runs of the procedure

each day, participant gaze position was calibrated using a 9-point visual gaze sequence. Additionally, prior to the first run of the experiment, corneal and pupil thresholds for reflectivity were determined. To stabilize head positions, participants performed the task in a chinrest set 55 cm away from the EyeLink camera. Additional details of the EyeLink data acquisition procedures can be found in our prior work[2].

Data Processing

Raw EyeLink data consisted of pupil diameter and x/y gaze position over time. We extracted three types of data from the EyeLink raw data: 1. pupil diameter, 2. blink rate, and 3. saccade rate. Data processing for the binocular acquired EyeLink data was initially applied to the both the left and right eyes independently. Subsequently, due to the greater quantity of subjects that had sufficient trials for the left eye compared to the right eye (N = 55 vs. N = 52), and very similar results for the two eyes, we used the left eye data for final analyses.

First, blinks and other artifacts were identified at the whole run level using the *Stublinks* procedure as described previously[2, 6]. For this procedure, data were initially downsampled from 1000 Hz to 60 Hz. *Stublinks* then identified blinks or other artifacts as pupil measurements fulfilling the following criteria: 1. diameter changes greater than 0.5 mm between consecutive samples, representing rapid changes typically associated with eyelid opening or closing; 2. Diameter values < 0.1 mm or values or more than ±4 mm from the median pupil diameter of the entire run, representing physiologically implausible pupil diameters; 3. Timepoints where the original 1000 Hz data and the 60 Hz downsampled data differed by >1mm; and 4. Samples with diameter outside of the Tukey's test interquartile range. Any period of consecutive timepoints

identified by the above criteria lasting 100-1000 ms was deemed a blink, and other consecutive periods <100ms or >1000ms were deemed other non-blink artifacts. Finally, all blink or other artifactual timepoints were removed from both the pupil diameter and eye gaze position time courses and linearly interpolated with adjacent non-artifactual samples.

Blink occurrence timecourses were generated by converting the *Stublinks*-identified blink samples into a binary vector where for each 1000Hz sample a value of 1 indicated the presence and a value of 0 indicated the absence of a blink. Saccade occurrence timecourses were determined by identifying timepoints in which gaze position subtended a portion of visual field > 1 degree and < 7 degrees of visual angle[7]. As with blink data, a binary vector containing all timepoints was then generated (1 = saccade, 0 = non-saccade) to represent the timepoints with presence of saccades in the run.

Following initial processing of raw data, pupil diameters were z-scored relative to the mean and standard deviation of all timepoints during gameplay for each run (quiz timepoints were excluded due to greater luminance during the quiz). Z-scoring was necessary to account for the variability in background luminance of different videos used for each run.

We next extracted 4 second time epochs centered around the confirmation of an action (-2 s before to +2 s after) for analysis. These trial epochs were identified with Ethernet timing messages communicated from the behavioral laptop (see Timing Synchronization section below). To identify and reject any trial epochs with eye tracker signal loss, we calculated the Pearson correlation between the pupil data and a straight line drawn from the first and last data

point in the epoch, and rejected any trials with r-value >0.99. Time courses for z-scored pupil data, blink rates, and saccade rates were then obtained by averaging all included aware and unaware trials separately first within each participant, and then calculating the mean and SEM across participants (e.g. **Fig S21 A-C**).

EEG

Equipment and Software

EEG data were collected with high density Ag/AgCl electrode nets (Hydrocel GSN 256, Magstim EGI Inc), sampled at 1000 Hz. Two systems were used: 1. Two Net Amps 200 128-Channel Amplifiers (256 channels total), and 2. One Net Amps 400 256-Channel Amplifier. Recordings were made on a 1. desktop computer (Power Mac G5 Quad; Mac OS X v10.5.8, Apple, Inc.) running NetStation 4.2.2 and 2. (A Macbook Pro 2018; MacOsX 10.14.2). Signals were acquired as Cz-referenced.

Data Preprocessing

EEG data were preprocessed with EEGLAB (function names in parentheses) at the session and the individual epoch level using a standard approach as described previously[2, 8]. Session level processing began with an initial 1 Hz high-pass filter to correct for drift (function *clean_drifts*). Next, line noise was removed from the data (function *pop_cleanline*). Noisy channels were next rejected (function *clean_channels*). Following noisy channel removal, the data were spherically interpolated (function *pop_interp*) and then re-referenced to the common average reference.

For event-related potential analysis, epochs of 4000 ms (2000 ms prior to action and 2000 ms following) were isolated from the data. Noisy data timepoints were found (function *clean_windows* with parameters -Inf, 7, and 0.25) and trials were then rejected if the quantity of noisy timepoints between 200 ms pre-action and 500 ms following action exceeded 175 ms (i.e. 25% of timepoints). The kept trials were then concatenated into a single vector (*channels* x *timepoints* x *epochs*). This vector was passed through a 10-component principal component analysis (PCA) for dimensionality reduction, and then through independent component analysis (ICA) upon the PCA data. Components corresponding to blink, saccade, cardiac, and myographic artifacts were identified by eye and rejected, and the remaining components recomposed to form the final participant data.

Time-frequency maps were extracted using wavelet decomposition (continuous wavelet transform; CWT) and individual epoch rejection was performed with short-time Fourier transform (STFT). Wavelet transformation was chosen as it demonstrated superiority in balancing temporal and frequency precision[9]. STFT was chosen for epoch rejection for ease of identifying high-power, artifactual frequencies. For these analyses, 6000 ms data vectors (3 seconds pre-action to 3 seconds post-action) were extracted and preprocessed as described above for event-related potentials. For the calculation of STFT, individual epochs were first divided into bins of 125 ms duration with 25% overlap. STFT was then performed in MATLAB extracting spectral power for 1-150 Hz (with function *spectrogram*) on each channel. These were then squared to give power values. Next, bins corresponding to timepoints 2 seconds pre-action to 1 second pre-action were identified for baselining and mean and standard deviation of power values calculated. The time interval of -2 to -1 seconds pre-action was chosen for baselining to

avoid any activity related to completion of the move sequence, which on average took less than ~500ms. All time bins were then z-scored using these values. The beta and gamma power z-score for each time bin was then calculated by averaging the z-score value of each constituent frequency of the band (12-30 Hz for beta, 40-140 Hz for gamma). If any time bin in any channel of the epoch contained an average beta power z-score value greater than 100, or average gamma power z-score value greater than 100, the trial was excluded.

For statistical analysis and visualization, CWT was performed on the included 6000 ms epochs. The implementation of the CWT involved convolving the data with a Morlet wavelet with frequency-dependent properties. We implemented convolution as a multiplication operation in the frequency domain. First, a discrete Fast Fourier transform was performed on each individual trial (using the MATLAB function *fft*). Next, for each frequency we analyzed (1-125 Hz), a Morlet wavelet was generated and a Fast Fourier transform was performed on it as well (using the MATLAB function *fft*). These two transformed vectors were then multiplied. An inverse discrete Fourier transform was then performed on the data (using the MATLAB function *ifft*) to return it to the time domain. These data were then converted to power values by squaring. For z-scoring each epoch, channel, and frequency, we used the timepoints from 2 seconds to 1 second prior to the action as baseline, and took the mean and standard deviation of these timepoints. The data timepoints for each epoch, channel and frequency were then z-scored using to the corresponding mean and standard deviation values.

Timing Synchronization

EEG

Task and behavioral events were transmitted from the behavioral laptop to an Arduino Uno R3 board via USB to deliver transistor-transistor logic pulses to the EEG amplifier (**Supplementary Fig S1**). For the Net Amps 200 system, the pulse was delivered from Arduino to amplifier with a DB9 cable. For the Net Amps 400 system, the pulse was either delivered via DB9 to a clock box which connected to the amplifier via MRTJ cable, or via DB9 to a HyperGrip adapter. Four events were employed for synchronization: 1. The answering of any quiz question, or late multiple choice message display; 2. Any visual state change (i.e. board disappearance, puzzle completion, quiz display); 3. Confirmation of the action with the spacebar, and 4. Selection of the block with the mouse. Timing testing with a photodiode on the behavioral laptop display was employed as described previously[1]. For the Net Amps 200 system and clock box connection, the photodiode signal appeared 70 ms after the arrival of the TTL pulse; and for the hypergrip cable, the photodiode reflex appeared 78 ms prior to the TTL pulse. Thus, a 148 ms difference was noted between the two connections. Therefore, epochs acquired from each system were extracted from raw data with these offsets corrected during the analysis.

EyeLink

Behavioral performance and eye measurements were synchronized via Ethernet messages sent between the experimental laptop and EyeLink PC (SR Research, Inc.)(**Supplementary Fig S1**). These messages included timing data on block selection, block movement, action confirmation, quiz display, and quiz answers. In addition to timing details, Ethernet messages also detailed the block selected, movement direction, quiz accuracy, and confidence slider submission position.

Statistical Analysis

Unless otherwise indicated all statistical analyses were done by pooling data first within each participant by averaging all trials of a given type (aware or unaware), and then comparing aware and unaware data across participants, so that the final analysis sample size was number of participants for a given data type.

Behavioral Analysis

Following behavioral data acquisition, *.psydat* files containing behavioral data were read into MATLAB (R2019, *Mathworks*) to extract raw data from the *.psydat* files. Designations for "aware" and "unaware" actions based upon quiz answers contained in the *.psydat* files were defined at the session level (six runs performed on a single day of testing). The multiple choice answers on the quiz (**Fig 1A**) were graded as correct or incorrect, and aggregated across all six runs in the session. Raw confidence values ranged from -450 (least confident) to +450 (most confident) based on the 900-pixel span centered on the screen (**Fig 1A**), and were also aggregated across all six runs. Raw confidence values for each session were then sorted by percentile. Any correct answer on the quiz followed by high confidence (>75th percentile within the session's data) was marked "Aware", and any incorrect answer followed by low confidence (<25th percentile) was marked "Unaware" (**Fig 1C-E**). These designations were then used to sort aware and unaware epochs for analysis of the EyeLink and EEG data.

Effects of participant demographics and game characteristics on behavioral responses were assessed using four key metrics: accuracy, confidence, awareness, and unawareness. These four metrics were calculated across runs (**Fig S21E; Supplementary Tables S4 and S5**), participants (**Supplementary Figs S2 and S4**; **Supplementary Tables S1, S2**), or day of testing

(**Supplementary Table S3**) depending on the context. Accuracy was defined as the total proportion of correctly answered questions on the multiple choice quiz (**Fig 1A**). Confidence was defined as the average confidence percentile across quizzes. Awareness was defined as the proportion of actions designated aware across quizzes. Unawareness was defined as the proportion of actions designated unaware across quizzes.

To analyze changes in accuracy, confidence, awareness, and unawareness between runs we performed two-tailed, paired t-tests comparing runs 2-5 pairwise with run 1, combining data across the two testing days within-participants ($p < 0.05$, Benjamini-Hochberg false-discovery rate correction; **Fig S21E**). To analyze differences in awareness, unawareness, confidence, and accuracy between days of testing we used two-tailed paired t-tests to compare values between days (**Supplementary Table S3**). To test awareness rates for each quiz timepoint (e.g. quiz *n-6* prior to an aware quiz was compared against quiz *n-6* prior to an unaware quiz) we used two-tailed paired t-tests, and the twelve time bins were corrected for significance with the Benjamini-Hochberg procedure ($p<0.05$; **Supplementary Fig S22**). Quiz timepoint barcharts were made by importing cell vector data from MATLAB to spreadsheet form, and then to GraphPad Prism 9. To compare differences in awareness, unawareness, confidence, and accuracy between block identity (red versus white), we first averaged the metric within each participant and block identity, then performed two-tailed, paired t-tests with Benjamini-Hochberg correction ($p < 0.05$; **Supplementary Table S1**). To compare differences in sex for these metrics across participants we used two-tailed, two-sample, t-tests with Benjamini-Hochberg correction ($p < 0.05$; **Supplementary Table S2**). Spearman correlation coefficients followed by two-tailed t-tests ($p<0.05$) were used to test for relationships between age versus awareness, unawareness,

confidence or accuracy across participants (**Supplementary Fig S2**). Spearman correlation coefficients followed by two-tailed t-tests ($p<0.05$) were used to test for relationships between age, video engagement or video familiarity versus awareness, unawareness, confidence, and accuracy across participants (**Supplementary Fig S2; Supplementary Tables S4 and S5**). Age-based plots were made by importing cell vector data from MATLAB to spreadsheet form, and then to GraphPad Prism 9.

To analyze the effects of the length of the delay between the confirmation of the action and the termination of the quiz, we sorted aware and unaware quizzes by delay time into 8 unique bins, from 2-8 seconds (**Supplementary Figure S4**). Mean subject awareness and unawareness correlation coefficients were performed in MATLAB by calculating the Pearson correlation between awareness or unawareness rates of an individual subject and bin duration, then averaging across subjects.

To analyze the impact of puzzle difficulty on key metrics of awareness, unawareness, accuracy, and confidence, we determined the initial puzzle configuration of the puzzle which corresponded to a quizzed move by extracting move data from *.psydat* files. The ten unique initial puzzle configurations could be solved in a minimum of 4-8 moves. We correlated the length of the shortest solution with awareness, unawareness, accuracy, and confidence of moves performed in those configurations.

EEG Spatiotemporal Analyses

For event-related potential analysis (**Figures 2, 3; Supplementary Figures S5-20**), 1000 Hz voltage data first underwent preprocessing (see above). Prior to statistical analysis, data epochs from -2s before to +2s after moves were then averaged within participants to obtain mean voltage timecourses for all aware and unaware trials for each electrode in each participant. Similarly, for power spectral analysis (**Fig 4**), 125ms Z-scored power data samples were first obtained through preprocessing (see above). Again, prior to statistical analysis data epochs from -2s before to +2s after moves were averaged within participants and within frequency bands of interest (alpha, 8 to 12 Hz; beta, 12 to 30 Hz; theta 4 to 8 Hz) to obtain mean timecourses for all aware and unaware trials for each electrode in each participant.

To correct for multiple comparisons, we performed spatiotemporal cluster-based permutation analyses on our data as previously described [2, 4, 10, 11]. In this analysis, an aggregate null distribution is constructed by randomly permuting our EEG data and identifying significant timepoints spatially or temporally adjacent to one another, which form spatiotemporal clusters. The same basic approach was performed separately for event-related potential data sampled at 1000 Hz, and for power spectral data with 125 ms samples.The following conditions were tested for statistically significant spatiotemporal clusters: 1. to identify significant changes in aware trials we tested aware data versus baseline; 2. to identify significant changes in unware trials we tested unaware data versus baseline; 3. to identify significant differences between aware and unaware trials we tested aware minus unaware data versus baseline. Each analysis began by creating an aggregate spatiotemporal null distribution using 5000 permutations. For each iteration, baseline data values (mean of 2 seconds pre-action to 1 second before action) were shuffled with test period data values at each time point from 1 second pre-action to 2 seconds

post-action. Each individual timepoint and electrode was tested for significance with a paired two-tailed t-test and significance level $p < 0.05$. To form clusters, spatial adjacency was determined by statistically significant channels in the same direction (positive or negative) neighboring each other in the electrode skull cap. Temporal adjacency was determined if any two or more timepoints were also deemed significant in the same direction. Following identification of spatiotemporal clusters, the summed absolute t-value (negative and positive clusters were identified separately) was calculated for all clusters. The cluster with the most positive and most negative t-value was then added to the respective aggregate null distribution. After constructing the null distribution, the cluster forming analysis described above was repeated on the non-permuted original data, and any cluster within the top 5% of the aggregate null distribution was considered to be statistically significant. To avoid spurious small clusters, we also required a minimum cluster size of 20 elements (sum of all electrodes in the cluster across consecutive time points) and duration of 20 ms for event-related potential analyses, and a minimum clusters size of 10 elements and duration of 200 ms for frequency power z-score analyses. Topoplots were constructed using EEGLAB's *topoplot* function to display voltage values for event-related potentials (**Figs 2, 3; Supplementary Fig S5-20**) or power z-score values for spectral analyses (**Fig 4**) at representative time points for significant clusters, with significant electrodes indicated by black outlines. Timecourse data for aware and unaware trials averaged across participants were also shown from representative electrodes within significant clusters, with a red line above the traces indicating contiguous time points with significant differences in the aware minus unaware condition from the spatiotemporal cluster analysis for a minimum duration of 20ms (to prevent spurious transient signals in single electrodes; **Figs 2-4; Supplementary Fig S5-20**).

EEG Robustness Analyses

To confirm that our results were robust to potential low trial counts and to correct for trial count imbalances between the aware and unaware conditions within participant, we performed a bootstrapping analysis on our data. Within each participant, we first identified whether there was an imbalance in aware and unaware trial counts. From the condition with a higher trial count, we randomly sampled (with replacement) trials equivalent to the number of lower trial counts, and generated a mean ERP trace across trials. We performed this sampling 1,000 times, creating 1,000 mean ERP traces. These 1,000 mean ERP traces were then averaged to create the new data for each participant used in the permutation analyses described above (**Supplementary Figures S19-S20**).

EyeLink Temporal Analyses

Time course data for. pupil diameter, blink rate, and saccade rate for aware and unaware trials were obtained as described above (see Eye Tracking and Pupillometry; Data Processing). To identify statistically significant differences between aware and unaware trials, we performed cluster based permutation using the same approach as in the preceding section but on temporal lines only. Again, to identify significant differences between aware and unaware trials we tested aware minus unaware data versus baseline. As with spatiotemporal cluster permutation, an aggregate temporal null distribution was initially generated with 5000 iterations. For each iteration, baseline data (the average value of all timepoints 2 seconds pre-action to 1 second before action) were permuted with test period data (1 second pre-action to 2 seconds post-action). The permuted data were then tested with a two-tailed paired t-test, and as with spatiotemporal clustering, clusters were identified by temporal adjacency (i.e. statistically

significant timepoints occurring in sequence), and negative and positive clusters were considered separately. Within cluster, t-values were summed and the cluster with the most positive and most negative t-value sum was then added to the respective aggregate null distributions. Clusters were then identified on the original unpermuted data with the same method as the permuted data (t-test compared to baseline mean), and then tested against the aggregate null distribution for significance ($p<0.05$). Any time points with significant differences between aware and unaware data were then indicated with a red line over the traces (**Fig S21 A-C**).

Pupil Run-by-Run Data

Run-by-run calculation of pupil data was performed in a similar manner as for run-by-run behavioral metrics (**Fig S21D**). Within individual aware and unaware epochs, the mean of all pupil diameter values one second prior to action until action performance was taken. These were then averaged within participant, combining corresponding runs across the two testing days, and then analyzed across participants ($N = 56$). One-sample t-tests were then used to compare runs 2-6 against the pupil diameter of run 1 ($p < 0.05$, Benjamini-Hochberg false-discovery rate correction).

Supplemental References

1. Herman, W.X., et al., *A Switch and Wave of Neuronal Activity in the Cerebral Cortex During the First Second of Conscious Perception.* Cereb Cortex, 2019. **29**(2): p. 461-474.

2. Kronemer, S.I., et al., *Human visual consciousness involves large scale cortical and subcortical networks independent of task report and eye movement activity.* Nat Commun, 2022. **13**(1): p. 7342.

3. Christison-Lagay, K.L., et al., *The neural activity of auditory conscious perception.* Neuroimage, 2025. **308**: p. 121041.

4. Gusso, M.M., et al., *More than a feeling: Scalp EEG and eye signals in conscious tactile perception.* Conscious Cogn, 2022. **105**: p. 103411.

5. Blumenfeld, H., *Brain Mechanisms of Conscious Awareness: Detect, Pulse, Switch, and Wave.* Neuroscientist, 2023. **29**(1): p. 9-18.

6. Siegle, G.J., et al., *Use of concurrent pupil dilation assessment to inform interpretation and analysis of fMRI data.* Neuroimage, 2003. **20**(1): p. 114-24.

7. Enderle, J., *12 - PHYSIOLOGICAL MODELING*, in *Introduction to Biomedical Engineering (Second Edition)*, J.D. Enderle, S.M. Blanchard, and J.D. Bronzino, Editors. 2005, Academic Press: Boston. p. 693-798.

8. Delorme, A. and S. Makeig, *EEGLAB: an open source toolbox for analysis of single-trial EEG dynamics including independent component analysis.* J Neurosci Methods, 2004. **134**(1): p. 9-21.

9. Kumar, N., Alam, K., and Siddiqi, A. H., *Wavelet Transform for Classification of EEG Signal using SVM and ANN.* Biomedical & Pharmacology Journal, 2017. **10**(4): p. 2061-2019.

10. Groppe, D.M., T.P. Urbach, and M. Kutas, *Mass univariate analysis of event-related brain potentials/fields I: a critical tutorial review.* Psychophysiology, 2011. **48**(12): p. 1711-25.

11. Nichols, T.E. and A.P. Holmes, *Nonparametric permutation tests for functional neuroimaging: a primer with examples.* Hum Brain Mapp, 2002. **15**(1): p. 1-25.
